# Maternal diabetes induces autism-like behavior by hyperglycemia-mediated persistent oxidative stress and suppression of superoxide dismutase 2

**DOI:** 10.1073/pnas.1912625116

**Published:** 2019-11-04

**Authors:** Xiumin Wang, Jianping Lu, Weiguo Xie, Xiaoyun Lu, Yujie Liang, Min Li, Zichen Wang, Xiaodong Huang, Mingxi Tang, Donald W. Pfaff, Ya-Ping Tang, Paul Yao

**Affiliations:** ^a^Joint Center of Translational Precision Medicine, Guangzhou Institute of Pediatrics, Guangzhou Women and Children Medical Center, 510623 Guangzhou, China;; ^b^Department of Child Psychiatry, Kangning Hospital of Shenzhen, 518020 Shenzhen, People’s Republic of China;; ^c^Institute of Rehabilitation Center, Tongren Hospital of Wuhan University, 430060 Wuhan, People’s Republic of China;; ^d^Department of Pathology, Affiliated Hospital of Southwest Medical University, Luzhou, 646000 Sichuan, China;; ^e^Laboratory of Neurobiology and Behavior, The Rockefeller University, New York, NY 10065;; ^f^Department of Imaging, Affiliated Hospital 3, Zhengzhou University, 450052 Zhengzhou, China

**Keywords:** autism spectrum disorders, epigenetics, oxidative stress, maternal diabetes, SOD2

## Abstract

Epidemiological studies show that maternal diabetes is associated with an increased risk of autism spectrum disorders (ASDs), although the detailed mechanisms remain unclear. The present study aims to investigate the potential effect of maternal diabetes on autism-like behavior in offspring. The results of in vitro study showed that transient hyperglycemia induces persistent reactive oxygen species (ROS) generation with suppressed superoxide dismutase 2 (SOD2) expression. Additionally, we found that SOD2 suppression is due to oxidative stress-mediated histone methylation and the subsequent dissociation of early growth response 1 (Egr1) on the SOD2 promoter. Furthermore, in vivo rat experiments showed that maternal diabetes induces SOD2 suppression in the amygdala, resulting in autism-like behavior in offspring. SOD2 overexpression restores, while SOD2 knockdown mimics, this effect, indicating that oxidative stress and SOD2 expression play important roles in maternal diabetes-induced autism-like behavior in offspring, while prenatal and postnatal treatment using antioxidants permeable to the blood–brain barrier partly ameliorated this effect. We conclude that maternal diabetes induces autism-like behavior through hyperglycemia-mediated persistent oxidative stress and SOD2 suppression. Here we report a potential mechanism for maternal diabetes-induced ASD.

## Significance

Hyperglycemia induces persistent oxidative stress and superoxide dismutase 2 (SOD2) suppression in neurons. SOD2 suppression is caused by oxidative stress-mediated histone methylation and subsequent dissociation of Egr1 on the SOD2 promoter. Maternal diabetes induces autism-like behavior in offspring with SOD2 suppression in the amygdala in rats, while SOD2 overexpression in the amygdala ameliorates autism-like behavior. Postnatal treatment of the blood–brain barrier-permeable antioxidant resveratrol partly restores this effect. This study describes a potential mechanism for maternal diabetes-induced autism-like behavior in offspring.

Autism spectrum disorders (ASDs) comprise a class of neurodevelopmental disorders characterized by deficits in social communication and interaction in addition to restricted, repetitive patterns of behavior, interests, or activities (1). Many factors, including genetics/epigenetics, sex, and environmental factors may contribute to ASD development; therefore, the complex etiology of ASD still requires further elucidation (2–5). Recent estimates of the prevalence of ASDs are around 1:59, with a male:female ratio of about 4:1 (4, 6).

Prenatal exposure to metabolic disturbance is associated with increased prevalence of ASDs and other neurodevelopmental disorders (7). Recent epidemiological studies clearly show that maternal diabetes, including type 1 diabetes (T1D), type 2 diabetes (T2D), and gestational diabetes mellitus (GDM) diagnosed by 26 wk, is associated with ASDs (8). T1D is considered to be the strongest factor (7, 9, 10), while the detailed mechanism for maternal diabetes-mediated ASDs remains unclear (11).

It has been reported that transient hyperglycemia induces persistent epigenetic changes and altered gene expression during subsequent normoglycemia (12), and that maternal diabetes is associated with epigenetic changes and neurodevelopmental impairment (13, 14). We presume that maternal diabetes may trigger a similar effect on the fetus, causing persistent epigenetic changes during neural development and resulting in an increased risk for ASDs.

We concentrated on estrogen signaling because of a long history of work on estrogens and prosocial behaviors. Importantly, dysregulation of estrogen receptor β (ERβ) has been associated with ASDs (15–18), and ERβ regulates SOD2 basal expression (19). Furthermore, estrogenic signaling in the amygdala has been implicated in mechanisms for social recognition and social memory. In fact, we have previously reported that prenatal progestin exposure induces suppression of ERβ and SOD2 in the amygdala and results in reactive oxygen species (ROS) formation and mitochondrial dysfunction, subsequently triggering autism-like behavior (3, 17). This indicates that the expression of SOD2 and ERβ in the amygdala may contribute to ASD development.

In this study, we aim to investigate the potential mechanisms for maternal diabetes-mediated ASD. We found that transient hyperglycemia exposure induces persistent oxidative stress and superoxide dismutase 2 (SOD2) suppression in in vitro cell experiments. Further investigation showed that hyperglycemia-induced SOD2 suppression is due to persistent oxidative stress-mediated histone methylation and the subsequent decreased association of Egr1 on the SOD2 promoter. SOD2 suppression is maintained during subsequent normoglycemia. The in vivo rat experiments showed that maternal T1D-induced autism-like behavior (ALB) in offspring can be restored by SOD2 overexpression in the amygdala. Furthermore, prenatal treatment of antioxidants, including resveratrol (RSV) (3, 20) and the SOD mimetic MnTBAP (21), can partly restore maternal diabetes-induced ALB. Postnatal treatment of these antioxidants showed that RSV can still partly restore this effect, while MnTBAP showed no significant effect. We conclude that maternal diabetes induces autism-like behavior through hyperglycemia-mediated persistent oxidative stress and SOD2 suppression.

## Results

### Hyperglycemia Induces SOD2 Suppression and Oxidative Stress.

**Fig. 1.**
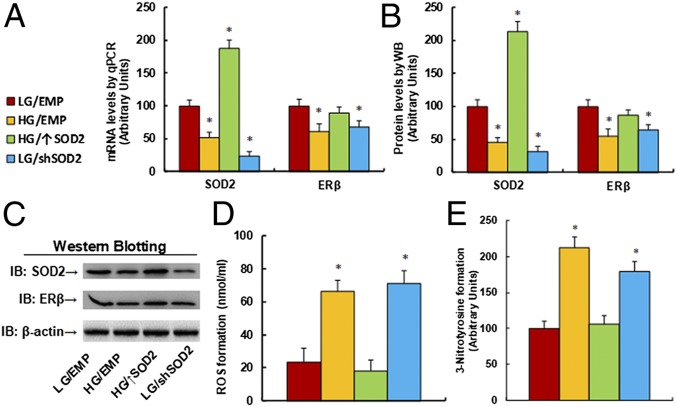
Hyperglycemia induces SOD2 suppression and oxidative stress. Human ACS-5003 neurons were infected with empty (EMP), SOD2 overexpression (↑SOD2), or SOD2 knockdown (shSOD2) lentivirus for 1 d and then treated with either 5 mM low glucose (LG) or 25 mM high glucose (HG) for 4 d in the presence of 1% serum; the cells were then harvested for further analysis. (*A*) The mRNA levels for gene expression of SOD2 and ERβ (*n* = 4). (*B*) Quantitation of protein levels (*n* = 5). (*C*) Representative Western blotting pictures for *B*. (*D*) ROS formation (*n* = 5). (*E*) The 3-nitrotyrosine formation (*n* = 5; *P < 0.05 vs. LG/EMP group). Data are expressed as mean ± SEM.

We first evaluated the effect of hyperglycemia on the gene expression of SOD2 and ERβ. Human ACS-5003 neurons were manipulated by either SOD2 overexpression (↑SOD2), knockdown (shSOD2), or empty lentivirus (EMP) for 1 d and were then exposed to either low glucose (LG, 5 mM) or high glucose (HG, 25 mM) for 4 d. Our results showed that HG treatment significantly decreased mRNA levels of SOD2 and ERβ to 51% and 61%, respectively, compared to the LG group. SOD2 overexpression lentivirus increased SOD2 mRNA to 187% and restored HG-induced ERβ suppression. In addition, SOD2 knockdown decreased SOD2 mRNA levels to 23%, but also decreased ERβ mRNA levels to 68% (Fig. 1*A*). We then measured the protein levels for those genes, and an expression pattern similar to that of the mRNA levels was observed (Fig. 1 *B* and *C* and *SI Appendix*, Fig. S1*A*). The results indicate that HG treatment suppresses the expression of SOD2 and ERβ and that ERβ expression seems to be regulated by SOD2. Finally, we measured hyperglycemia-induced oxidative stress in neurons (Fig. 1 *D* and *E*). The results showed that hyperglycemia (HG) increased reactive oxygen species (ROS) formation (Fig. 1*D*) and 3-nitrotyrosine formation (Fig. 1*E*) to 286% and 212%, respectively, compared to the LG group. Furthermore, SOD2 overexpression (↑SOD2) completely restored, while SOD2 knockdown (shSOD2) mimicked, this effect.

### Hyperglycemia Induces SOD2 Suppression through Histone Methylation and the Subsequent Decreased Association of Egr1 on the SOD2 Promoter.

**Fig. 2.**
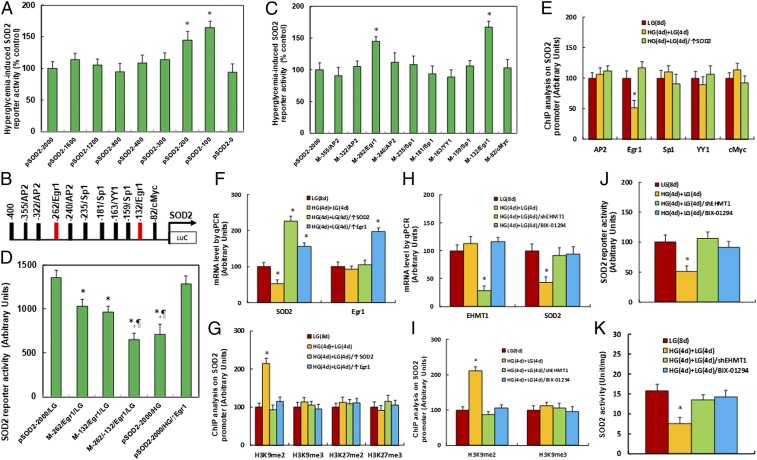
Hyperglycemia induces SOD2 suppression through histone methylation and the subsequent decreased association of Egr1 on the SOD2 promoter. (*A*) The conditional immortalized ACS-5003 neurons were transiently transfected by either SOD2 full length (pSOD2-2000) or deletion reporter plasmids. After 24 h, the cells were treated by either 5 mM low glucose (LG) or 25 mM high glucose (HG) for 3 d, and the SOD2 reporter activities were calculated (*n* = 4; *P < 0.05 vs. pSOD2-2000 group). (*B*) The schematic picture for the potential transcriptional binding motif in the range of approximately −400 to 0 (from transcription start site) on the SOD2 promoter in addition to the 2 potential Egr1 binding sites are marked in red. (*C*) The cells were transiently transfected by either wild type SOD2 reporter construct (pSOD2-2000) or single point mutation at the site shown in *B* and then treated with either LG or HG for 3 d, and the SOD2 reporter activities were calculated (*n* = 4; *P < 0.05 vs. pSOD2-2000 group). (*D*) The cells were transiently transfected by SOD2 full length (pSOD2-2000) or single point mutation as indicated or infected by Egr1 lentivirus (↑Egr1), and were then treated with either LG or HG for 3 d; the SOD2 reporter activities were then calculated (*n* = 4; *P < 0.05 vs. pSOD2-2000/LG group; ^¶^*P* < 0.05 vs. M-262/Egr1/LG group). (*E*) Cells were treated by either 8-d LG [LG(8 d)], or 4-d HG plus 4-d LG [HG(4 d)+LG(4 d)] or the cells were infected on day 4 by SOD2 lentivirus [HG(4 d)+LG(4 d)/SOD2↑]; the cells were then used for ChIP analysis [*n* = 4; *P < 0.05 vs. LG(8 d) group]. (*F* and *G*) Cells were treated by either LG(8 d) or HG(4 d)+LG(4 d) or the cells were infected on day 4 by SOD2 [HG(4 d)+LG(4 d)/↑SOD2] or Egr1 lentivirus [HG(4 d)+LG(4 d)/↑Egr1]; the cells were then used for ChIP analysis [*n* = 4; *P < 0.05 vs. LG(8 d) group]. (*H*–*K*) Cells were treated by either LG(8 d) or HG(4 d)+LG(4 d), and the cells were infected on day 4 by shEHMT1 lentivirus [HG(4 d)+LG(4 d)/shEHMT1] or cells were treated on day 4 by EHMT1 inhibitor BIX-01294 [HG(4 d)+LG(4 d)/BIX-01294]; then, cells were used for the following further analysis: (*H*) mRNA analysis (*n* = 4), (*I*) ChIP analysis by H3K9me2 and H3K9me3 antibodies (*n* = 4), (*J*) SOD2 reporter activity assay (*n* = 5), and (*K*) SOD2 activities [*n* = 5; *P < 0.05 vs. LG(8 d) group]. Data are expressed as mean ± SEM.

We investigated the potential molecular mechanism for hyperglycemia-induced SOD2 suppression. A series of progressive 5′-promoter deletion constructs for the SOD2 promoter were generated, and these constructs were transfected into conditional immortalized neurons for the analysis of SOD2 reporter activity in the presence of either 5 mM LG or 25 mM HG for 24 h. We found that hyperglycemia-induced SOD2 reporter suppression occurred among the −2,000, −1,600, −1,200, −800, −400, −300, and −0 deletion constructs (numbered according to Ensembl gene ID SOD2-201 ENST00000337404.8; transcription start site was marked as 0), while suppression was significantly restored in the −200 and −100 deletion reporter constructs, indicating that hyperglycemia-responsive transcriptional element is located in the range of approximately −300 to −100 on the SOD2 promoter (Fig. 2*A*). The transcription factor databases TESS revealed many potential binding motifs, including 3 of AP2, 2 of Egr1 (marked in red; see Fig. 2*B*), 3 of Sp1, and 1 of YY1 and cMyc binding sites located in the range of approximately −300 to −100 on the SOD2 promoter (Fig. 2*B*). We then mutated these potential binding motifs in the SOD2 full length (pSOD2-2000) reporter construct, and the reporter assay showed that hyperglycemia-induced SOD2 suppression did not occur on the Egr1 binding motif mutation located at −262 and −132, respectively (Fig. 2*C*). We then made double mutations on both of the Egr1 binding sites in the pSOD2 full length construct (M-262/-132/Egr1), and the reporter assay showed that Egr1 double mutation significantly decreased SOD2 reporter activity in the LG treatment group compared to the wild type full length, while it mimicked the reporter activity of the full length reporter construct (pSOD2-2000) in the HG treatment (Fig. 2*D*). Our results indicate that hyperglycemia induces SOD2 expression through decreased association of Egr1 on the SOD2 promoter.

We then conducted ChIP analysis using antibodies for transcription factors of AP2, Egr1, Sp1, YY1, and cMyc, as shown in Fig. 2*B*. The cells were treated by HG for 4 d, then switched to LG for another 4 d [HG(4 d)+LG(4 d)]. The results showed that the binding ability of Egr1 on the SOD2 promoter was significantly decreased to 51% compared to the control group after 8 d in LG [LG(8 d)], and this effect was completely restored by infection of SOD2 on day 4 after the 4-d HG treatment [HG(4 d)+LG(4 d)/↑SOD2; Fig. 2*E*]. We further measured gene expression levels and found that SOD2 mRNA levels decreased to 53% after 4-d HG treatment followed by 4-d LG treatment [HG(4 d)+LG(4 d)] compared to the LG(8 d) treatment. This effect was completely restored by infection of either SOD2 or Egr1 [HG(4 d)+LG(4 d)/↑Egr1] on day 4 after the 4-d HG treatment. On the contrary, the expression of Egr1 did not change during either LG or HG treatment (Fig. 2*F*). Given the fact that Egr1 expression levels did not change during hyperglycemia treatment, it can be suggested that hyperglycemia-induced decreased association of Egr1 on the SOD2 promoter may be due to hyperglycemia-induced epigenetic changes in the range of approximately −300 to 100 on the SOD2 promoter. We first evaluated histone acetylation on the SOD2 promoter using the acetyl-histone H4 (K5, K8, K12, K16) antibody that recognizes histone H4 acetylated at lysines 5, 8, 12, or 16 and the acetyl-histone H3 (K9, K14, K18, K23, K27) antibody that recognizes histone H3 acetylated at lysines 9, 14, 18, 23, or 27 by ChIP analysis (*SI Appendix*, Fig. S2*A*). The results showed that there was no significant difference in either histone H3 or H4 acetylation.

We then measured histone methylation on the SOD2 promoter. We first evaluated histone H4 methylation on the SOD2 promoter (*SI Appendix*, Fig. S2*B*) and found that hyperglycemia did not have any effect on histone H4 methylation. We then evaluated the effect of hyperglycemia on histone H3 methylation (Fig. 2*G*). The results showed that hyperglycemia treatment had no effect on the methylation of H3K9me3, H3K27me2, and H3K27me3, while methylation of H3K9me2 increased to 215% as a result of HG(4 d)+LG(4 d) treatment compared to LG(8 d) treatment; on the contrary, infection of either SOD2 [HG(4 d)+LG(4 d)/↑SOD2] or Egr1 [HG(4 d)+LG(4 d)/↑Egr1] completely restored this effect. In order to further confirm that SOD2 suppression is due to hyperglycemia-induced H3K9me2 modification on the SOD2 promoter, the neurons were treated by either shRNA lentivirus for EHMT1, a specific histone methyltransferase that is responsible for H3K9me2 methylation (22), or an EHMT1-specific inhibitor BIX-01294, and the cells were harvested for further analysis (Fig. 2 *H*–*K*). We first measured mRNA expression. The results showed that shEHMT1 infection [HG(4 d)+LG(4 d)/shEHMT1] decreased EHMT1 mRNA expression to 28% compared to the LG(8 d) group, indicating a successful knockdown by the shEHMT1 lentivirus. EHMT1 inhibitor BIX-01294 [HG(4 d)+LG(4 d)/BIX-01294] had no effect on EHMT1 expression, while both the shEHMT1 lentivirus and BIX-01294 completely restored hyperglycemia-induced SOD2 suppression (Fig. 2*H*). We then measured histone methylation on the SOD2 promoter using ChIP analysis. The results showed that both shEHMT1 and BIX-01294 completely restored hyperglycemia-induced H3K9me2 modification (Fig. 2*I*). Finally, we measured the SOD2 reporter (Fig. 2*J*) and SOD2 enzyme activity (Fig. 2*K*). The results showed that both shEHMT1 and BIX-01294 completely restored hyperglycemia-induced decreased SOD2 reporter activity and enzyme activity. Our results indicate that hyperglycemia-induced SOD2 suppression in neurons is due to hyperglycemia-induced H3K9me2 histone methylation and the subsequent dissociation of Egr1 on the SOD2 promoter.

### Hyperglycemia-Induced Persistent Oxidative Stress and SOD2 Suppression Were Restored by Overexpression of Either SOD2 or Egr1.

**Fig. 3.**
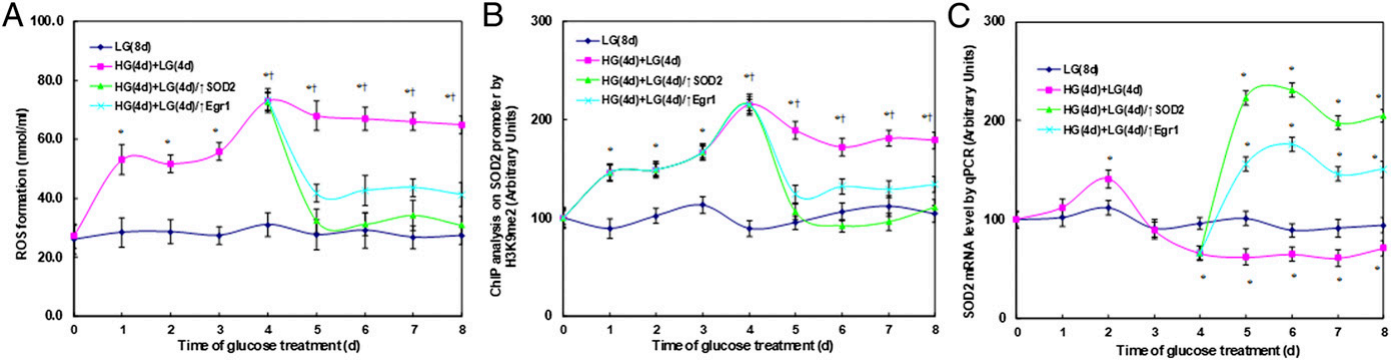
Hyperglycemia-induced persistent oxidative stress and SOD2 suppression were restored by expression of SOD2/Egr1 lentivirus. The human ACS-5003 neurons were treated by either 8-d LG [LG(8 d)] or 4-d HG plus 4-d LG [HG(4 d)+LG(4 d)] or the cells were infected on day 4 by SOD2 lentivirus [HG(4 d)+LG(4 d)/↑SOD2] or Egr1 lentivirus [HG(4 d)+LG(4 d)/↑Egr1]; the cells were then used for further analysis: (*A*) ROS formation (*n* = 4), (*B*) ChIP analysis on SOD2 promoter (*n* = 4), and (*C*) SOD2 mRNA levels [*n* = 4; *P < 0.05 vs. LG(8 d) at day 0 group; ^¶^*P* < 0.05 vs. LG(8 d) at day 1 group]. Data are expressed as mean ± SEM.

We first measured the effect of hyperglycemia on ROS generation in ACS-5003 neurons (Fig. 3*A*). The neurons were first exposed to high glucose (25 mM) for 4 d, then switched to low glucose (5 mM) for an additional 4 d. We found that ROS generation increased to 195% on day 1 and then stayed constant until day 3. ROS generation then significantly increased to 269% on day 4 compared to the original levels on day 0. On day 5, it decreased slightly but maintained high levels, with a 250% increase. These high levels of ROS lasted for 4 d, with a 239% increase (on day 8) compared to day 0. On the contrary, hyperglycemia-induced persistent elevated ROS generation was completely diminished after infection of either SOD2 lentivirus (↑SOD2) or Egr1 lentivirus (↑Egr1). We measured hyperglycemia-induced histone modification H3K9me2 on the SOD2 promoter (Fig. 3*B*) and SOD2 mRNA expression (Fig. 3*C*). The results showed that both SOD2 (↑SOD2) and Egr1 (↑Egr1) lentivirus completely restored hyperglycemia-induced H3K9me2 modification and SOD2 suppression. Our results indicate that hyperglycemia-induced persistent epigenetic changes with H3K9me2 and subsequent SOD2 suppression may be due to hyperglycemia-induced persistent oxidative stress.

### Maternal Diabetes Induces Suppression of SOD2 and ERβ with Oxidative Stress and Mitochondrial Dysfunction, while SOD2 Overexpression Restores, and SOD2 Knockdown Mimics, This Effect.

**Fig. 4.**
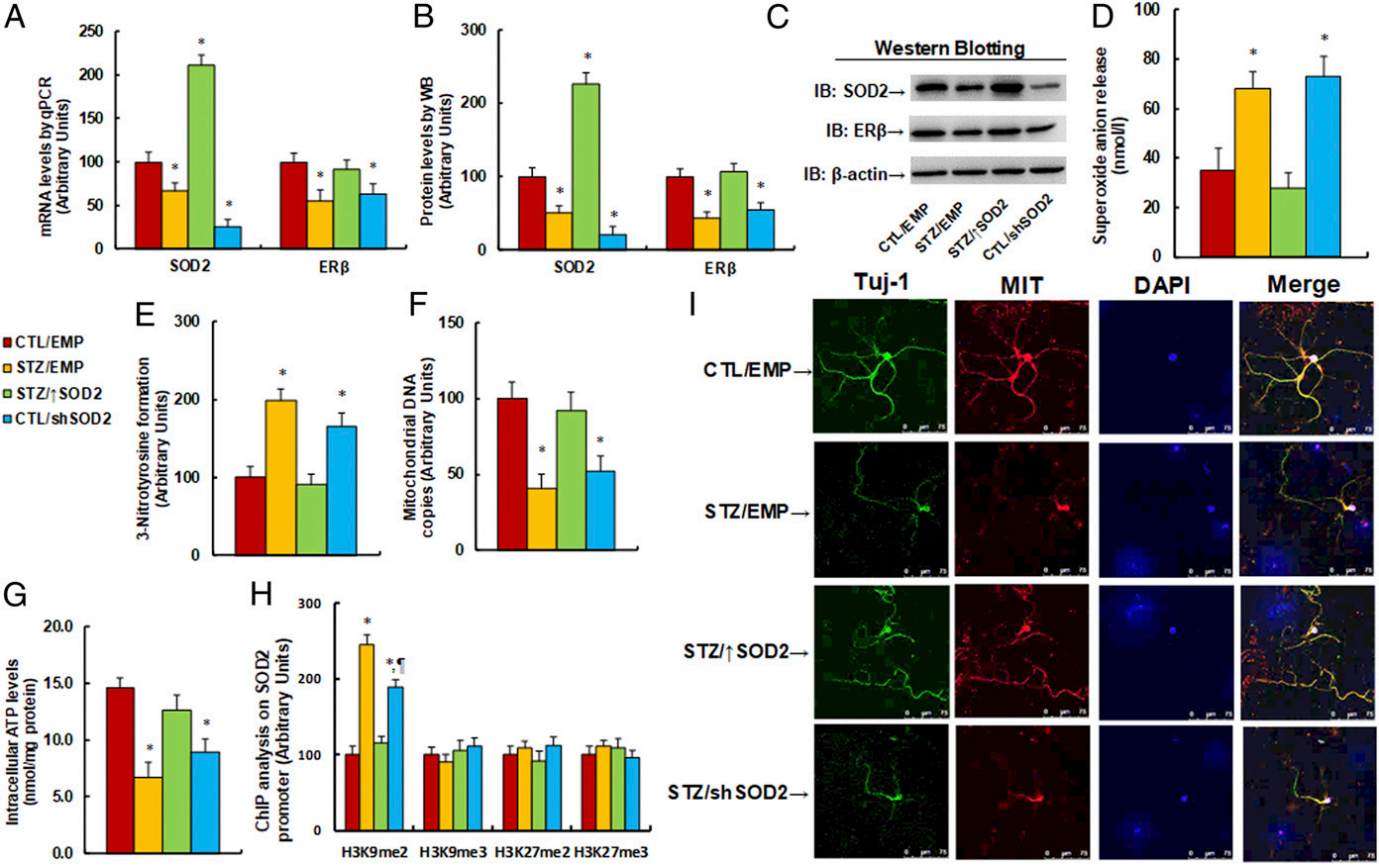
Maternal diabetes induces suppression of SOD2 and ERβ with oxidative stress and mitochondrial dysfunction, while SOD2 overexpression restores, and SOD2 knockdown mimics, this effect. The 6-wk-old male offspring from dams where diabetes (STZ) had been induced or from controls (CTL) received empty (EMP), SOD2 overexpression (↑SOD2), or SOD2 knockdown (shERβ) lentivirus infusion; then, the offspring at 8 wk of age were sacrificed for further analysis. (*A*–*J*) The amygdala tissues were isolated for further analysis as below: (*A*) mRNA levels for gene expression of SOD2 and ER (*n* = 4), (*B*) representative pictures for Western blotting, (*C*) quantitation of protein levels (*n* = 5), (*D*) in vivo superoxide anion release (*n* = 5), (*E*) quantitation of 3-nitrotyrosine formation (*n* = 5), (*F*) mitochondrial DNA copies (*n* = 4), and (*G*) intracellular ATP levels (*n* = 5). (*H* and *I*) The amygdala neurons were isolated at embryonic day (E18) from the above treatment for further analysis. (*H*) ChIP analysis for histone modification in amygdala neurons (*n* = 4). (*I*) Immunostaining in amygdala neurons. The Tuj-1 was stained as neuron marker (green), mitochondria (MIT) was stained by MitoBeacon (red), nuclei were stained by DAPI (blue), and “merge” means the mixed color of triple staining (*P < 0.05 vs. CTL/EMP group; ^¶^*P* < 0.05 vs. STZ/EMP group). Data are expressed as mean ± SEM. (Magnification, 400×.)

The 6-wk-old male offspring came from dams where diabetes (STZ) had been induced or from controls (CTL). The offspring received empty (EMP), SOD2 overexpression (↑SOD2), or knockdown (shSOD2) lentivirus infusion to the amygdala, and were later sacrificed at 8 wk of age for analysis of gene expression and subsequent molecular consequences in the amygdala. Our results showed that maternal diabetes exposure decreased SOD2 mRNA to 67% compared to the CTL group, and that SOD2 overexpression (↑SOD2) increased, while SOD2 knockdown (shSOD2) decreased, SOD2 mRNA to 211% and 26%, respectively, indicating a successful SOD2 expression manipulation by infusion of the lentivirus in the amygdala. We also measured the ERβ mRNA levels. The results showed that prenatal STZ exposure decreased ERβ mRNA levels to 55%, and ↑SOD2 treatment restored, while shSOD2 treatment mimicked, the effect (Fig. 4*A*). We then measured the protein levels of these genes, and an expression pattern similar to that of the mRNA levels was observed (Fig. 4 *B* and *C* and *SI Appendix*, Fig. S1*B*). We then measured the molecular consequences of maternal diabetes exposure-mediated gene expression. Our results showed that STZ/EMP treatment significantly increased ROS formation, including superoxide anion release (Fig. 4*D*) and 3-nitrotyrosine formation (Fig. 4*E*). Overexpression of SOD2 (STZ/↑SOD2) completely restored this effect compared to the CTL/EMP control group, while SOD2 knockdown (CTL/shSOD2 group) mimicked this effect.

**Fig. 5.**
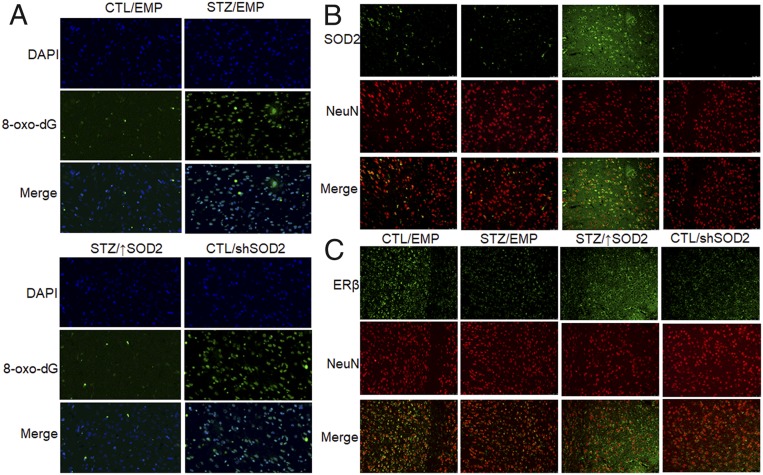
Immunohistochemistry staining of amygdala. The 6-wk-old male offspring from either the control (CTL) or maternal diabetes (STZ) groups received empty control (EMP), SOD2 overexpression (↑SOD2), or SOD2 knockdown (shERβ) lentivirus infusion. Then, the offspring at 8 wk old were sacrificed, and the amygdala was isolated for immunohistochemistry staining. (*A*) The 8-oxo-dG staining for oxidative stress (green) and DAPI staining for nuclei (blue). (*B*) SOD2 staining (green) and NeuN staining for neurons (red). (*C*) ERβ staining (green) and NeuN staining for neurons (red). (Magnification, 100×.)

We then measured the effect of maternal diabetes exposure on mitochondrial function. Our results showed that maternal diabetes exposure (STZ/EMP) significantly decreased mitochondrial DNA copies (Fig. 4*F*) and intracellular ATP levels (Fig. 4*G*) compared to the CTL/EMP control group. Again, SOD2 expression (STZ/↑SOD2 group) completely restored, and SOD2 knockdown (CTL/shSOD2 group) mimicked, the effect. In addition, we measured the maternal diabetes-induced epigenetic changes on the SOD2 promoter in isolated amygdala neurons (Fig. 4*H*). We found that maternal diabetes treatment increased H3K4me2 epigenetic modification to 245% compared to the CTL/EMP group. SOD2 expression completely restored, while SOD2 knockdown only partly mimicked, the effect. We also evaluated the effect of maternal diabetes on the mitochondria mass by immunostaining in isolated amygdala neurons (Fig. 4*I*). The results showed that STZ/EMP treatment had significantly less mitochondria staining compared to the CTL/EMP group, and SOD2 expression (STZ↑SOD2) completely restored, while SOD2 knockdown (CTL/shSOD2) mimicked, this effect. Finally, we evaluated oxidative stress and gene expression in the amygdala using immunohistochemistry staining. Staining with 8-oxo-dG was used as the marker of oxidative stress (Fig. 5*A* and *SI Appendix*, Fig. S3*A*), and the STZ/EMP significantly increased 8-oxo-dG staining to 316% compared to the CTL/EMP group. Again, SOD2 expression (STZ/↑SOD2 group) completely restored, and SOD2 knockdown (CTL/shSOD2 group) mimicked, the effect of diabetes. Additionally, the proteins for SOD2 (Fig. 5*B* and *SI Appendix*, Fig. S3*B*) and ERβ (Fig. 5*C* and *SI Appendix*, Fig. S3*B*) were stained and quantitated. The results showed that STZ/EMP treatment significantly decreased expression of SOD2 and ERβ to 42% and 61%, respectively, compared to the CTL/EMP control group. SOD2 expression (STZ/↑SOD2 group) increased expression of SOD2 and ERβ to 348% and 236%, respectively, while SOD2 knockdown (CTL/shSOD2 group) decreased expression of SOD2 and ERβ to 16% and 37%, respectively, compared to the CTL/EMP group.

In addition, we measured gene expression in the hypothalamus (*SI Appendix*, Fig. S4*A*) and hippocampus (*SI Appendix*, Fig. S4*B*). Gene expression of SOD2 and ERβ showed no significant difference in either the hypothalamus or the hippocampus. Our results indicate that maternal diabetes-induced oxidative stress and mitochondrial dysfunction is due to hyperglycemia-induced SOD2 suppression.

### Maternal Diabetes Induces Autism-Like Behavior in Offspring, while SOD2 Overexpression Partly Restores, and SOD2 Knockdown Mimics, This Effect.

**Fig. 6.**
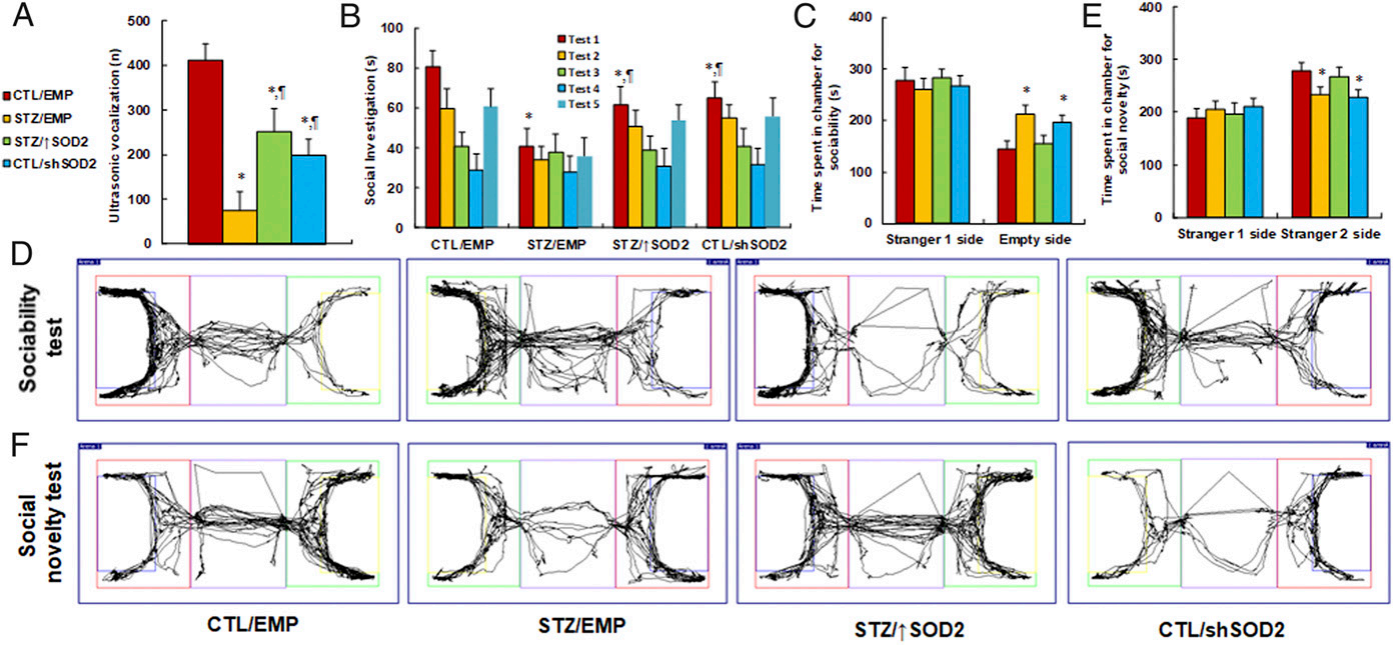
Maternal diabetes induces autism-like behavior in offspring, while SOD2 overexpression restores, and SOD2 knockdown mimics, this effect. The 6-wk-old male offspring from either the control (CTL) or maternal diabetes (STZ) groups received empty control (EMP), SOD2 overexpression (↑SOD2), or SOD2 knockdown (shERβ) lentivirus infusion, and then the offspring at 8 wk of age were used for autism-like behavior analysis. (*A*) Ultrasonic vocalization (*n* = 9). (*B*) Social recognition: seconds socially investigating a conspecific [same conspecific in tests 1 to 4; novel conspecific in test 5 (a new stimulus rat was introduced), *n* = 9; *P < 0.05 vs. CTL/EMP group; ^¶^*P* < 0.05 vs. STZ/EMP group]. (*C*–*F*) Three-chambered social tests (*n* = 8). (*C*) Time spent in chamber for sociability. (*D*) Representative trace recorder for *C*. (*E*) Time spent in chamber for social novelty. (*F*) Representative trace recorder for *E* (*P < 0.05 vs. CTL/EMP group; ^¶^*P* < 0.05 vs. STZ/EMP group). Data are expressed as mean ± SEM.

We continued to evaluate the effect of maternal diabetes on autism-like behaviors with the manipulation of SOD2 expression. We first measured the ultrasonic vocalization. The results showed that ultrasonic vocalization frequency (Fig. 6*A*) decreased to 13% in the maternal diabetes group (STZ/EMP) compared to the CTL/EMP group. SOD2 overexpression in the maternal diabetes group (STZ/↑SOD2) partly restored this effect, while SOD2 knockdown in control offspring (CTL/shSOD2) partly mimicked it. We then evaluated the effect using social recognition tests. Social recognition significantly differed between 4 treatments [*F*(3,32) = 2.622, *P* = 0.038]. Subsequent post hoc analysis revealed that habituation to the same stimulus conspecific (tests 1 to 4) was significant in the CTL/EMP group [*F*(3,32) = 4.591, *P* < 0.01], STZ/↑SOD2 group [*F*(3,32) = 3.468, *P* < 0.041], and CTL/shSOD2 group [*F*(3,32) = 3.614, *P* < 0.039], but not in the STZ/EMP group. Dishabituation was significant in the CTL/EMP group [*F*(1,8) = 4.253, *P* < 0.01], and borderline significant in the STZ/↑SOD2 group [*F*(1,8) = 2.734, *P* = 0.039] and CTL/shSOD2 group [*F*(1, 8) = 2.427, *P* = 0.044], but was not significant in the STZ/EMP group.

We further measured the effect using 3-chambered social tests. The results showed that STZ/EMP treatment increased time spent in the empty side of the chamber for sociability (Fig. 6 *C* and *D*) to 146% and decreased time spent in the empty side of the chamber for social novelty (Fig. 6 *E* and *F*) to 84%, respectively, compared to the CTL/EMP group. Furthermore, STZ/↑SOD2 treatment completely restored this effect, and CTL/shSOD2 completely mimicked it. In addition, there was no difference in the stranger 1 side in terms of either sociability or social novelty. Our results indicate that maternal diabetes-induced autism-like behavior can be partly restored by postnatal expression of SOD2 in the amygdala.

### Prenatal Treatment of SOD Mimetic MnTBAP and Resveratrol Partly Ameliorates Maternal Diabetes-Induced Autism-Like Behavior in Offspring.

**Fig. 7.**
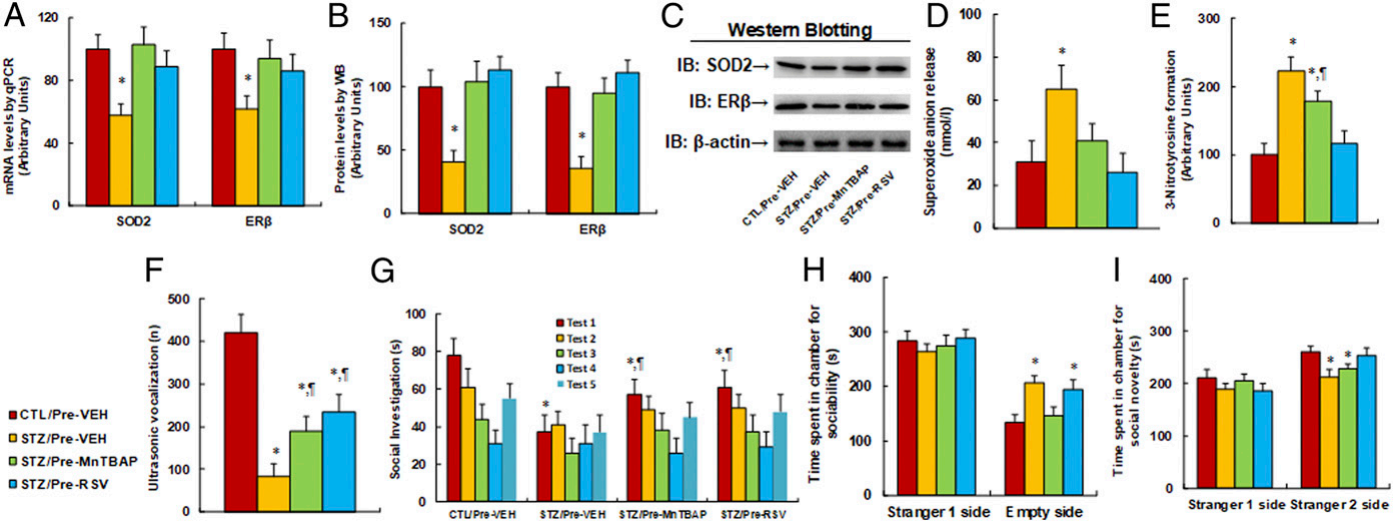
Prenatal treatment of SOD mimetic MnTBAP and resveratrol partly ameliorates maternal diabetes-induced autism-like behavior in offspring. The dams from either maternal diabetes (STZ) or control (CTL) group received treatments of either SOD mimetic MnTBAP or resveratrol (RSV), and the 8-wk-old male offspring were used for autism-like behavior analysis before being sacrificed for further biomedical analysis. (*A*–*E*) The amygdala tissues were isolated for further analysis as below: (*A*) mRNA levels for gene expression of SOD2 and ERβ (*n* = 4), (*B*) representative pictures for Western blotting, (*C*) quantitation of protein levels (*n* = 5), (*D*) in vivo superoxide anion release (*n* = 5), (*E*) quantitation of 3-nitrotyrosine formation (*n* = 5), (*F*–*I*) autism-like behavior analysis (*n* = 8), and (*F*) ultrasonic vocalization (*n* = 9). (*G*) Social recognition: seconds socially investigating a conspecific [same conspecific in tests 1 to 4; novel conspecific in test 5 (a new stimulus rat was introduced), *n* = 9; *P < 0.05 vs. CTL/EMP group; ^¶^*P* < 0.05 vs. STZ/EMP group]. (*H* and *I*) Three-chambered social tests for time spent in chamber for sociability (*H*) and time spent in chamber for social novelty (*I*). *P < 0.05 vs. CTL/Pre-VEH group. Data are expressed as mean ± SEM.

We evaluated the potential effect of prenatal treatment of SOD mimetic MnTBAP and resveratrol on autism-like behavior. The dams from either the maternal diabetes (STZ) or control (CTL) group received treatments of either SOD mimetic MnTBAP or resveratrol (RSV), and the 8-wk-old male offspring were used for autism-like behavior analysis before they were sacrificed for further biomedical analysis. We first evaluated gene expression, and we found that the maternal diabetes group (STZ/Pre-VEH) decreased mRNA expression of SOD2 and ERβ to 58% and 62%, respectively, compared to the control (CTL/Pre-VEH) group, and that prenatal treatment of either SOD mimetic (STZ/Pre-MnTBAP) or resveratrol (STZ/Pre-RSV) completely restored this effect (Fig. 7*A*). We also measured the protein levels of those genes, and a pattern similar to that of the mRNA levels was observed (Fig. 7 *B* and *C* and *SI Appendix*, Fig. S1*C*). We then measured the effect on oxidative stress. The results showed that the STZ/Pre-VEH group increased superoxide anion release (Fig. 7*D*) and 3-nitrotyrosine formation (Fig. 7*E*) to 210% and 223%, respectively, compared to the CTL/Pre-VEH group, and that prenatal treatment of MnTBAP partly, while treatment of RSV completely, restored this effect.

Afterward, we evaluated the effect of prenatal treatment of the antioxidants on autism-like behaviors. The results showed that STZ/EMP treatment decreased ultrasonic vocalization frequency (Fig. 7*F*) to 16% compared to the CTL/Pre-VEH group. Prenatal treatment of either STZ/Pre-MnTBAP or STZ/Pre-RSV partly restored this effect, and RSV had a stronger effect than MnTBAP. We also evaluated the effect using social recognition tests. Social recognition significantly differed between 4 treatments [*F*(3,32) = 2.816, *P* = 0.042]. Subsequent post hoc analysis revealed that habituation to the same stimulus conspecific (tests 1 to 4) was significant in the CTL/Pre-VEH group [*F*(3,32) = 4.684, *P* < 0.01], STZ/Pre-MnTBAP group [*F*(3,32) = 3.561, *P* < 0.046], and STZ/Pre-RSV group [*F*(3,32) = 3.726, *P* < 0.037], but not in the STZ/Pre-VEH group. Dishabituation was significant in the CTL/Pre-VEH group [*F*(1,8) = 4.694, *P* < 0.01] and borderline significant in the STZ/Pre-MnTBAP [*F*(1,8) = 2.954, *P* = 0.043] and STZ/Pre-RSV groups [*F*(1,8) = 2.937, *P* = 0.040], but not significant in the STZ/Pre-VEH group. We then evaluated the effect of prenatal treatment of antioxidants using 3-chambered social tests. The results showed that the STZ/Pre-VEH group increased time spent in the empty side of the chamber for sociability (Fig. 7*H*) to 154% and decreased time spent in the empty side of the chamber for social novelty (Fig. 7*I*) to 89%, respectively, compared to the CTL/Pre-VEH group. Furthermore, the STZ/Pre-RSV group completely restored this effect, while STZ/Pre-MnTBAP had little effect. In addition, there was no difference in time spent in the stranger 1 side for both sociability and social novelty. Our results indicate that maternal diabetes-induced autism-like behavior can be partly restored by prenatal treatment of SOD mimetic MnTBAP and RSV.

### Postnatal Treatment of Resveratrol Partly Ameliorates Maternal Diabetes-Induced Autism-Like Behavior in Offspring, while SOD Mimetic MnTBAP Has No Effect.

**Fig. 8.**
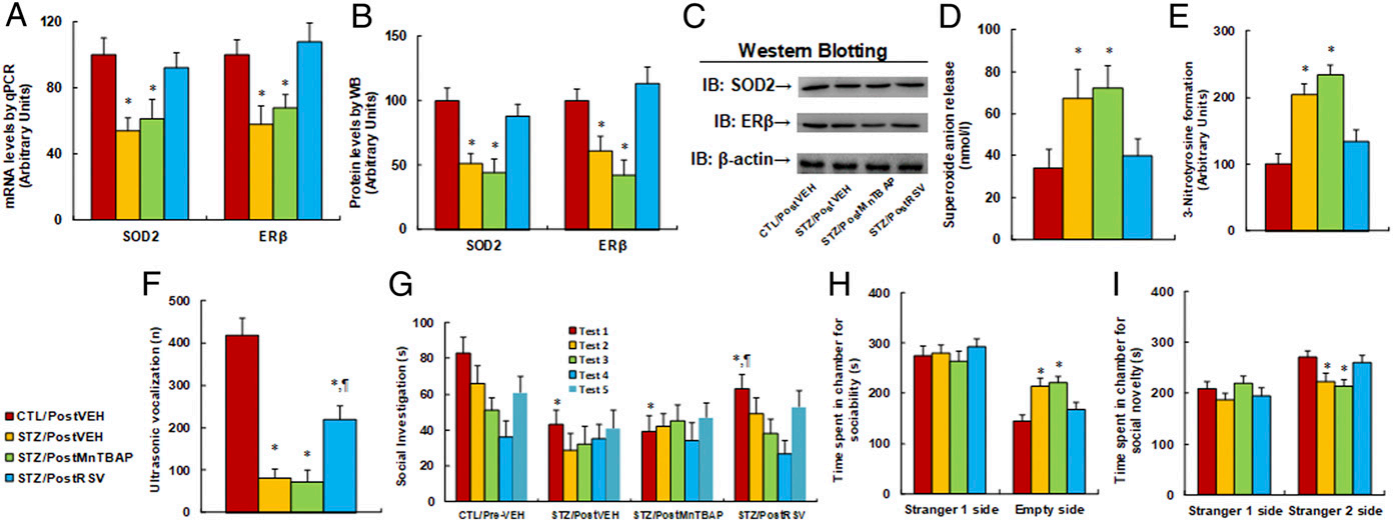
Postnatal treatment of resveratrol partly ameliorates maternal diabetes-induced autism-like behavior in offspring, while SOD mimetic MnTBAP has no effect. The male offspring at 6 wk old from either the maternal diabetes (STZ) or control (CTL) groups received treatments of either SOD mimetic MnTBAP or resveratrol (RSV), and the 8-wk-old male offspring were used for autism-like behavior analysis before being sacrificed for further biomedical analysis. (*A*–*E*) The amygdala tissues were isolated for further analysis as below: (*A*) mRNA levels for gene expression of SOD2 and ERβ (*n* = 4), (*B*) representative pictures for Western blotting, (*C*) quantitation of protein levels (*n* = 5), (*D*) in vivo superoxide anion release (*n* = 5), (*E*) quantitation of 3-nitrotyrosine formation (*n* = 5), (*F*–*I*) autism-like behavior analysis (*n* = 8), and (*F*) ultrasonic vocalization (*n* = 6). (*G*) Social recognition: seconds socially investigating a conspecific [same conspecific in tests 1 to 4; novel conspecific in test 5 (a new stimulus rat was introduced), *n* = 9; *P < 0.05 vs. CTL/EMP group; ^¶^*P* < 0.05 vs. STZ/EMP group]. (*H* and *I*) Three-chambered social tests for time spent in chamber for sociability (*H*) and time spent in chamber for social novelty (*I*). *P < 0.05 vs. CTL/Pre-VEH group. Data are expressed as mean ± SEM.

We evaluated the potential effect of postnatal treatment of SOD mimetic MnTBAP and resveratrol on autism-like behavior. The male offspring at 6 wk old from either the maternal diabetes (STZ) or control (CTL) group received treatments of either SOD mimetic MnTBAP or resveratrol (RSV), and the 8-wk-old male offspring were used for autism-like behavior analysis before they were sacrificed for further biomedical analysis. We first evaluated the levels of gene expression. We found that the maternal diabetes (STZ/Pre-VEH) group had mRNA expression levels of SOD2 and ERβ that decreased to 54% and 58%, respectively, compared to the control (CTL/PostVEH) group, and postnatal treatment of SOD mimetic (STZ/PostMnTBAP) had no effect, while postnatal treatment of resveratrol (STZ/PostRSV) completely restored this effect (Fig. 8*A*). We also measured the protein levels of these genes, and a pattern similar to that of mRNA levels was observed (Fig. 8 *B* and *C* and *SI Appendix*, Fig. S1*D*). We then measured the effect on oxidative stress. The results showed that STZ/Pre-VEH treatment increased superoxide anion release (Fig. 8*D*) and 3-nitrotyrosine formation (Fig. 8*E*) to 197% and 204%, respectively, compared to the CTL/Pre-VEH group, and postnatal treatment of MnTBAP had no effect, while RSV completely restored this effect. We proceeded to evaluate the effect of postnatal treatment of these antioxidants on autism-like behaviors. The results showed that the STZ/PostVEH group had a decreased ultrasonic vocalization frequency (Fig. 8*F*) to 14% compared to the CTL/PostVEH group. Postnatal treatment of MnTBAP (STZ/PostMnTBAP) had no effect, while postnatal treatment of RSV (STZ/PostRSV) partly restored this effect.

We also evaluated the effect using social recognition tests. Social recognition significantly differed between 4 treatments [*F*(3,32) = 3.012, *P* = 0.031]. Subsequent post hoc analysis revealed that habituation to the same stimulus conspecific (tests 1 to 4) was significant in the CTL/PostVEH group [*F*(3,32) = 4.521, *P* < 0.01] and STZ/PostRSV group [*F*(3,32) = 3.464, *P* < 0.038], but not in the STZ/Pre-VEH and TSZ/PostMnTBAP groups. Dishabituation was significant in the CTL/PostVEH group [*F*(1,8) = 4.427, *P* < 0.01] and STZ/PostRSV group [*F*(1,8) = 2.648, *P* = 0.046], but not significant in the STZ/PostVEH and STZ/PostMnTBAP groups. We then evaluated the effect of postnatal treatment of antioxidants using 3-chambered social tests. The results showed that, in the STZ/PostVEH group, time spent in the empty side of the chamber for sociability (Fig. 8*H*) increased to 148%, while time spent in the empty side of the chamber for social novelty (Fig. 8*I*) decreased to 82%, respectively, compared to the CTL/PostVEH group. Furthermore, STZ/PostRSV group completely restored this effect, while STZ/PostMnTBAP had no effect. In addition, there was no difference in time spent in the stranger 1 side for both sociability and social novelty. Our results indicate that maternal diabetes-induced autism-like behavior can be partly restored by postnatal treatment of RSV, but not SOD mimetic MnTBAP.

## Discussion

In this study, we demonstrated that transient hyperglycemia induces persistent oxidative stress with maintained SOD2 suppression during subsequent normoglycemia. Furthermore, we discovered that SOD2 suppression is due to oxidative stress-mediated histone methylation and subsequent dissociation of Egr1 on the SOD2 promoter in neurons. Maternal diabetes induces autism-like behavior in offspring, with suppressed expression of SOD2 and ERβ in the amygdala. SOD2 overexpression in the amygdala ameliorates autism-like behavior, while prenatal treatment of antioxidants MnTBAP and RSV partly prevents, and postnatal treatment of RSV partly restores, this effect.

### Hyperglycemia-Induced SOD2 Suppression.

The human neural progenitor cell ACS-5003 was used for in vitro study to investigate the potential effect of hyperglycemia on gene expression and epigenetic changes in neurons. ACS-5003 cells have the ability to differentiate into neurons during in vitro cell culture, which was used to mimic the process of development of stem cells into neurons during embryo development. We have obtained consistent results from both in vitro and in vivo study for hyperglycemia-induced epigenetic changes. We found that transient hyperglycemia exposure induces persistent SOD2 suppression during subsequent normoglycemia. Suppressed SOD2 expression results in further ROS generation with oxidative stress, forming a positive feed-forward loop for ROS generation (12). We also found that hyperglycemia-induced SOD2 suppression is due to oxidative stress-mediated histone methylation with H3K9me2 and the subsequently decreased binding ability of Egr1 on the SOD2 promoter. This indicates that hyperglycemia-induced persistent oxidative stress and epigenetic changes on the SOD2 promoter may be the potential driving force for damage in neurons and subsequently contribute to ASD development (23, 24). In addition, some other factors that are induced by maternal diabetes may also contribute to ASD development, such as hyperglycemia-induced persistent inflammation and suppression of the NFκB signaling pathway (12).

### Role of SOD2 Expression in ASD Development.

Our study showed that transient hyperglycemia induces persistent SOD2 suppression in cells. Furthermore, maternal diabetes induces SOD2 suppression in offspring with autism-like behavior. Overexpression of SOD2 partly restores this effect, indicating that SOD2 expression plays an important role in autism-like behavior. This is consistent with previous discoveries (25–27), although some other factors may also be involved in this process. For instance, expression of ERβ and ERRα has been found to play an important role in prenatal progestin exposure-induced autism-like behavior (3, 17). We also found that SOD2 suppression results in decreased ERβ expression, which subsequently triggers the dysfunction of mitochondrial and fatty acid metabolism that is associated with ASD development (3, 17, 28). This is consistent with previous findings from epidemiology and clinical studies (18, 28). In addition, male offspring were used for this study because previous studies have shown that male offspring are more likely to develop autism-like behavior than female offspring; a possible explanation is that basal ERβ expression in the amygdala is significantly higher in female rats than in male rats (3, 17).

### Role of Antioxidants in Maternal Diabetes-Induced ASD.

We showed that maternal diabetes-induced autism-like behavior is restored by SOD2 expression in the amygdala. Prenatal treatment of antioxidants SOD mimetic MnTBAP (21, 29) and RSV (3, 20) partly restored this effect, indicating that oxidative stress plays an important role in ASD development. Additionally, postnatal treatment of antioxidant RSV shows an effect similar to that of prenatal treatment, while postnatal treatment of MnTBAP has no effect, indicating that RSV may penetrate the blood–brain barrier while MnTBAP does not (3). In addition, RSV has many other functions besides its antioxidant action; it also blocks certain potassium channels in neurons and has many effects on the SIRT1, PPARs, and PGC1α pathways (30–34). Thus, the potential function of RSV on ASD could be complicated. On the contrary, the clinical application of RSV for ASD treatment faces obstacles due to its potential renal toxicity, and development of an effective antioxidant that can penetrate the blood–brain barrier is still a significant challenge.

## Conclusions

Maternal diabetes induces persistent oxidative stress and triggers epigenetic changes with histone methylation at H3K9me2 on the SOD2 promoter, subsequently resulting in SOD2 suppression with related embryo damage and autism-like behavior in offspring. We conclude that maternal diabetes may induce autism-like behavior, in part through hyperglycemia-mediated persistent oxidative stress and SOD2 suppression.

### Methods.

An expanded methods section is available in *SI Appendix*, *SI Methods*.

### In Vivo Rat Experiments.

#### Rat protocol 1 for generation of diabetic offspring.

Adult (3 mo old) female Sprague–Dawley rats were monitored for estrous cycles with daily vaginal smears. Only rats with at least 2 regular 4- to 5-d estrous cycles were included in the studies. Chronic diabetic female rats were induced by injection of 50 mg/kg streptozocin (STZ; 0.05 M sodium citrate, pH 5.5) after an 8-h fasting period. Animals with blood glucose >300 mg/dL were considered positive, while control (CTL) rats received only vehicle injection. The females were caged with proven males, and pregnancy was verified through observation of a sperm plug, which was designated as day 0 of pregnancy. The amygdala neurons were isolated on embryonic day 18 (E18) as described later. The male offspring were separated from the dams on day 21 and fed until 7 to 8 wk of age for further experiments. Some of the 7- to 8 wk-old offspring were then used for autism-like behavior tests. After that, the offspring were sacrificed, and various brain tissues, including the amygdala, hypothalamus, and hippocampus, were isolated, flash-frozen in dry ice, and then stored in a −80 °C freezer for immunohistochemistry and analysis of gene expression, SOD2 activity, superoxide anion release, DNA damage, and mitochondrial function.

#### Rat protocol 2 for postnatal manipulation of SOD2 expression.

The male offspring (6 wk old) from either the CTL or STZ group in rat protocol 1 were anesthetized with a mixture of ketamine (90 mg/kg) and xylazine (2.7 mg/kg) and implanted with a guide cannula targeting the amygdala (26 gauge; Plastics One) (35). The following coordinates were chosen for the amygdala: −2.0 mm posterior to bregma, ±4.2 mm from the midline, and −7.2 mm from the skull surface on which it was based. A cannula was attached to the skull with dental acrylic and jeweler’s screws and closed with an obturator (36). An osmotic minipump (model 2002; flow rate 0.5 μL/h; Alzet) connected to a 26-gauge internal cannula that extended 1 mm below the guide was implanted and used to deliver SOD2 overexpression (↑SOD2), SOD2 knockdown (shSOD2), or empty (EMP) lentivirus. Vehicle consisting of artificial cerebrospinal fluid (aCSF; 140 mM NaCl, 3 mM KCl, 1.2 mM Na_2_HPO_4_, 1 mM MgCl_2_, 0.27 mM NaH_2_PO_4_, 1.2 mMCaCl_2_, and 7.2 mM dextrose, pH 7.4) was used for the infusion of the lentivirus. Infusion (flow rate 0.5 µL/h) begun immediately after placement of the minipump. Lentivirus (0.5 μL of total 2 × 10^3^ cfu) was infused for 1 h. Rats received the infusion of lentivirus of either SOD2 knockdown (shSOD2) or overexpression (↑SOD2) or empty control (EMP). The experimental rats were separated into 4 groups (10 per group): group 1, CTL offspring with empty control lentivirus infusion (CTL/EMP); group 2, STZ offspring with empty control lentivirus infusion (LNG/EMP); group 3, STZ offspring with SOD2 expression lentivirus infusion (STZ/↑SOD2); and group 4, CTL offspring with SOD2 knockdown lentivirus infusion (VEH/shSOD2). Cannula placement was verified histologically postmortem by the injection of 0.5 μL of India ink (volume-matched drug delivery in the experiments). Rats whose dye injections were not located in the amygdala were excluded from the data analysis. At 2 wk after lentivirus infusion, the offspring were used for behavior tests followed by biomedical analysis, as indicated in rat protocol 1 (17).

#### Rat protocol 3 for prenatal treatment of antioxidants.

Verified pregnant dams from either the CTL or STZ group in rat protocol 1 were randomly assigned to the following 4 groups: group 1, CTL group rats received only s.c. vehicle (5% DMSO in maize oil) injection (CTL/PreVEH); group 2, diabetic (STZ) rats received only vehicle injection (STZ/PreVEH); group 3, diabetic (STZ) rats received 10 mg/kg/d of MnTBAP (dissolved in DMSO) injection (STZ/PreMnTBAP); and group 4, diabetic (STZ) rats received 20 mg/kg of resveratrol (RSV; dissolved in DMSO) injection (STZ/PreRSV). The injection was conducted on days 1, 4, 7, 10, 15, and 20 of pregnancy, respectively. The male offspring were separated from the dams on day 21 and fed until 7 to 8 wk old for behavior tests followed by biomedical analysis.

#### Rat protocol 4 for postnatal treatment of antioxidants.

The male offspring (3 wk old) from either the CTL or STZ group in rat protocol 1 were randomly assigned to the following 4 groups: group 1, CTL group rats received only s.c. vehicle injection (CTL/PostVEH); group 2, STZ group received only vehicle injection (STZ/PostVEH); group 3, STZ group received MnTBAP injection (STZ/PostMnTBAP); and group 4, STZ group received RSV injection (STZ/PostRSV). The injection was conducted once every 3 d and continuously for 4 wk, and the rats were sacrificed at 7 to 8 wk of age for behavior tests followed by biomedical analysis.

#### Animal behavior test.

The animal behavior test of offspring was carried out at 7 to 8 wk of age. Autism-like behavior was evaluated using ultrasonic vocalizations and a 3-chambered social test as described later (37–39).

#### Ultrasonic vocalizations (USVs).

The USVs of neonates were examined during brief maternal separation on postnatal day 7. USVs from individually isolated pups were recorded using an externally polarized condenser microphone with a frequency range of 30 to 300 kHz that was attached 15 to 20 cm above the floor of an isolation chamber. The microphone was connected to the Avisoft-UltrasoundGate recording software (Avisoft Bioacoustics), and the pup-emitted calls were recorded to WAV sound files using parameters optimized for rats. Pups were individually placed in the sound-proof chambers, and calls were recorded for 300 s. Data transformation on the number of USVs were analyzed using a generalized linear model with a negative binomial distribution and a log-link function (37, 38).

#### Social recognition.

Social recognition was defined by reduced time spent investigating a familiar conspecific as a result of social habituation, and subsequent reinstatement of investigation when a novel intruder is introduced (dishabituation). Unfamiliar age- and sex-matched intact stimulus rats were placed in wire mesh containers. Before the test, the stimulus rats were gently habituated to being in the container, and focal rats were habituated to having an empty container in their home cage. Each focal rat was tested 5 times (tests 1 to 5) in their home cage, in which a container with a stimulus rat was introduced. Each test lasted 5 min, and the tests were repeated with a 15-min interval. During the 15-min interval, the same empty container was placed back in the home cage of the focal rat. The same stimulus rat was used for the first 4 tests, whereas, for the fifth test, the stimulus rat was replaced with another unfamiliar sex- and age-matched conspecific. The placement of the containers over the 5 tests was kept constant. During the tests, the rats were left undisturbed, and their behavior was videotaped and subsequently scored using the JWatcher software program. Social investigation was defined as sniffing the wire mesh part of the container (38, 39).

#### Three-chambered social test.

Seven- to 8-wk-old rats were used to assess sociability and preference for social novelty. Target subjects (stranger 1 and stranger 2) were 7- to 8-wk-old rats habituated to being placed inside wire cages for 3 d prior to the beginning of testing, and test rats were habituated to the testing room for at least 45 min prior to the start of behavioral tasks. For the sociability test, the test animal was introduced to the middle chamber and left to habituate for 5 min, after which an unfamiliar mouse (stranger 1) was introduced into a wire cage in one of the side-chambers and an empty wire cage on the other side-chamber. The test animal was allowed to freely explore all 3 chambers over a 10-min session. Following this, a novel stranger rat (stranger 2) was introduced into the previously empty wire cage, and the test animal was again left to explore for a 10-min session. Parameters scored include time spent in each chamber and number of entries into the chambers. Time spent in each chamber and track maps were calculated using automated SMART software (39).

### Data Availability.

All data are available in the manuscript and *SI Appendix*.

## Supplementary Material

Supplementary File
