# Supplementary material for: Maternal diabetes induces autism-like behavior by hyperglycemia-mediated persistent oxidative stress and suppression of superoxide dismutase 2
Source: Proc Natl Acad Sci U S A. 2019 Nov 4;116(47):23743–52. doi: 10.1073/pnas.1912625116 (PMC6876200; doi:10.1073/pnas.1912625116)
Supplement: Supplementary File [file pnas.1912625116.sapp.pdf]

PNAS
www.pnas.org

Supplemental Information for

Maternal Diabetes Induces Autism-Like Behavior through Hyperglycemia-Mediated Persistent Oxidative Stress and Suppression of Superoxide Dismutase 2

Xiumin Wang, Jianping Lu, Weiguo Xie, Xiaoyun Lu, Yujie Liang, Min Li, Zichen Wang, Xiaodong Huang, Mingxi Tang, Donald W. Pfaff, Ya-Ping Tang, Paul Yao

Author correspondence:

Paul Yao, E-mail: vasilis112@yahoo.com

Ya-Ping Tang, E-mail: yptang12@126.com

Donald W. Pfaff, E-mail: pfaff@rockefeller.edu

This PDF file includes:

Supplementary text

Table S1

Figures S1 to S4

SI References

www.pnas.org/cgi/doi/10.1073/pnas.1912625116

SI METHODS

Reagents and materials. The Neural Progenitor Cell Origin ATCC-BXS0117 Normal; Human (ATCC# ACS-5003) was obtained from ATCC, and cultured in NPC medium, including 464mL DMEM: F12 (ATCC® 30-2006) supplemented with the Growth Kit for Neural Progenitor Cell Expansion (ATCC® ACS-3003) with the following components: 5 mL L-Alanyl-L-Glutamine; 5 mL Non-Essential Amino Acids; 10 mL NPC Growth Kit Component A; 5 mL NPC Growth Kit Component B; 1 mL NPC Growth Kit Component C; 10 mL NPC Growth Kit Component D, together with 100 U/ml penicillin, 100 μg/ml streptomycin and 10% human serum. All cells were maintained in a humidified incubator with 5% CO_2 at 37°C. In some experiments, the ACS-5003 neurons were conditionally immortalized using a hTERT lentivirus vector with an extended life span to achieve higher transfection efficiency and experimental stability (1, 2).

The antibodies for AP2α (sc-12726), β-actin (sc-47778), cMyc (sc-40), Egr1 (sc-515830), ERβ (sc-137381), SOD2 (sc-30080), Sp1 (sc-17824) and YY1 (sc-7341) were obtained from Santa Cruz Biotechnology. Antibodies for acetyl-histone H4 K5, K8, K12, and K16 (H4K5,8,12,16ac, #PA5-40084) were obtained from Invitrogen. Antibodies for anti-histone H3 acetyl K9, K14, K18, K23, K27(H3K9,14,18,23,27ac, ab47915), H4K20me1 (ab9051), H4K20me3 (ab9053), H4R3me1 (ab17339), H3K9me2 (ab1220), H3K9me3 (ab8898), H3K27me2 (ab24684) and H3K27me3 (ab6002), H2AX (ab20669) and γH2AX (ab2893) were obtained from Abcam, 3-nitrotyrosine (3-NT) was measured using the 3-Nitrotyrosine ELISA Kit (ab116691 from Abcam) per manufacturers' instructions. The mitochondrial fraction was isolated using a Pierce Mitochondria Isolation Kit (Pierce Biotechnology) per manufacturers' instructions. Nuclear extracts were prepared using the NE-PER Nuclear and Cytoplasmic Extraction Reagents Kit (Pierce Biotechnology). Protein concentration was measured using the Coomassie Protein Assay Kit (Pierce Biotechnology). Luciferase activity assay was carried out using the Dual-Luciferase™ Assay System (Promega) and the transfection efficiency was normalized using a cotransfected renilla plasmid (3). Resveratrol (RSV, #R5010), streptozocin (STZ, #18883-66-4), and MnTBAP, a cell-permeable superoxide dismutase (SOD) mimetic and peroxynitrite scavenger (#475870), were obtained from Sigma. H3K9m2 specific inhibitor BIX-01294 (sc-202651) was purchased from Santa Cruz Biotechnology.

Construction of SOD2 reporter plasmid. Human genomic DNA was prepared from NPC cells. In order to construct SOD2 reporter plasmids, the SOD2 gene promoter (2kb upstream of the transcription start site plus first exon) was amplified from Ensembl gene ID: SOD2 ENST00000337404 by PCR and subcloned into the pGL3-basic vector (# E1751, Promega) using restriction sites of Mlu I and Hind III with the following primers: SOD2 forward: 5'-gcgc-acgcgt- gaa tcc tgt gga ttc atc ctt -3' (Mlu I) and SOD2 reverse: 5'- gtac- aagctt- ctg aag acg aga aag cac agc -3' (Hind III). To map SOD2 promoter activity, the related deletion promoter constructs were generated by PCR methods and subcloned into the pGL3-basic vector. All the vectors were verified by sequencing, and detailed information on these plasmids is available upon request (3).

Generation of SOD2/ERβ/Egr1 expression lentivirus. The cDNA for human ERβ, SOD2 and Egr1, and rat SOD2 were obtained from Open Biosystems. The cDNA for either SOD2, ERβ, or Egr1 was subcloned into the pLVX-Puro vector (from Clontech) with the restriction sites of Xho1 and Xba1 using the below primers: human ERβ forward primer: 5'- gtac - ctcgag- atg gat ata aaa aac tca cca -3' (Xho1) and human ERβ reverse primer: 5'- gtac - tctaga- tca ctg ctc cat cgt tgc ttc -3' (Xba1); human SOD2 forward primer: 5'- gtac- ctcgag- atg ttg agc cgg gca gtg tgc -3' (Xho1) and human SOD2 reverse primer: 5'- gtac- tctaga- tta ctt ttt gca agc cat gta -3' (Xba1); human Egr1 forward primer: 5'- gtac - ctcgag- atg gcc gcg gcc aag gcc gag -3' (Xho1) and human Egr1

reverse primer: 5’- gtac - tctaga- tta gca aat ttc aat tgt cct -3’ (Xba1); rat SOD2 forward primer: 5’-gtac-ctcgag- atg ttg tgt cgg gcg gcg tgc -3’ (Xho1) and rat SOD2 reverse primer: 5’-gtac-tctaga-tca ctt ctt gca aac tat gta -3’ (Xba1). The lentivirus for SOD2/ERβ/Egr1 or empty control (CTL) was expressed through Lenti-X™ Lentiviral Expression Systems (from Clontech) per manufacturers’ instructions.

Gene knockdown by shRNA lentivirus particles. The shRNA lentivirus plasmids for human SOD2 (sc-41655-SH), ERβ (sc-35325-SH) and EHMT1 (sc-62261-SH) or rat SOD2 (sc-270084-SH), or non-target control (sc-108060) were purchased from Santa Cruz Biotechnology. The related lentivirus for genes of SOD2, ERβ, EHMT1 or empty control (CTL) were expressed through Lenti-X™ Lentiviral Expression Systems (from Clontech) per manufacturers’ instructions. The purified and condensed lentivirus were used for either in vitro (for human cells) or in vivo (for rat) gene knockdown. The knockdown efficiency was confirmed by more than 65% of mRNA reduction compared to the control group in cells using real time PCR (see Table S1).

RT reaction and real-time quantitative PCR. Total RNA from treated cells was extracted using the RNeasy Micro Kit (Qiagen), and the RNA was reverse transcribed using an Omniscript RT kit (Qiagen). All the primers were designed using Primer 3 Plus software with the Tm at 60°C, primer size of 21bp, and the product length in the range of 140-160bp (see Table S1). The primers were validated with the amplification efficiency in the range of 1.9-2.1, and the amplified products were confirmed with agarose gel. Real-time quantitative PCR was run on iCycler iQ (Bio-Rad) with the Quantitect SYBR green PCR kit (Qiagen). The PCR was performed by denaturing at 95°C for 8 min, followed by 45 cycles of denaturation at 95°C, annealing at 60°C, and extension at 72°C for 10s, respectively. 1 µl of each cDNA was used to measure target genes. β-actin was used as the housekeeping gene for transcript normalization, and the mean values were used to calculate relative transcript levels with the $^{\Delta\Delta}$CT method per instructions from Qiagen. In brief, the amplified transcripts were quantified by the comparative threshold cycle method using β-actin as a normalizer. Fold changes in gene mRNA expression were calculated as $2^{-\Delta\Delta CT}$ with CT = threshold cycle, ΔCT=CT (target gene)-CT(β-actin), and the ΔΔCT =ΔCT (experimental)-ΔCT (reference) (3, 4).

Western blotting. Cells were lysed in an ice-cold lysis buffer (0.137M NaCl, 2mM EDTA, 10% glycerol, 1% NP-40, 20mM Tris base, pH 8.0) with protease inhibitor cocktail (Sigma). The proteins were separated in 10% SDS-PAGE and further transferred to the PVDF membrane. The membrane was incubated with appropriate antibodies, washed and incubated with HRP-labeled secondary antibodies, and then the blots were visualized using the ECL+plus Western Blotting Detection System (Amersham). The blots were quantitated by IMAGEQUANT, and final results were normalized by β-actin (3, 4).

Luciferase reporter assay. 1.0×10^5 of treated cells were seeded in a 6-well plate with complete medium to grow until they reached 80% confluence. Cells were then cotransfected by 3µg of VEGF full length or deletion reporter constructs, together with 0.2µg of pRL-CMV-Luc *Renilla* plasmid (from Promega). Then, cells were treated by either 5mM aspirin or empty control (CTL) for 24 hours. After treatment, the cells were harvested and the luciferase activity assays were carried out using the Dual-Luciferase™ Assay System (Promega), and the transfection efficiencies were normalized using a cotransfected *Renilla* plasmid according to manufacturers’ instructions. The VEGF reporter activity from either the ASA or control (CTL) group was calculated (3).

Chromatin immunoprecipitation (ChIP). Cells were washed and crosslinked using 1% formaldehyde for 20 min and terminated by 0.1M glycine. Cell lysates were sonicated and

centrifuged. 500µg of protein were pre-cleared by BSA/salmon sperm DNA with preimmune IgG and a slurry of Protein A Agarose beads. Immunoprecipitations were performed with the indicated antibodies, BSA/salmon sperm DNA and a 50% slurry of Protein A agarose beads. Input and immunoprecipitates were washed and eluted, then incubated with 0.2mg/ml Proteinase K for 2h at 42˚C, followed by 6h at 65˚C to reverse the formaldehyde crosslinking. DNA fragments were recovered through phenol/chloroform extraction and ethanol precipitation. A ~150bp fragment in the range of -300~-100 from the transcription start site on the SOD2 promoter was amplified by real-time PCR (qPCR) using the primers provided in Table S1 (3, 4), and the amplified products were further confirmed by agarose gel.

Measurement of ROS generation. Treated cells were seeded in a 24-well plate and incubated with 10µM CM-H2DCFDA (Invitrogen) for 45 min at 37°C, and then the intracellular formation of reactive oxygen species (ROS) was measured at excitation/emission wavelengths of 485/530nm using a FLx800 microplate fluorescence reader (Bio-Tek). The data was normalized as arbitrary units (3, 5).

Measurement of DNA breaks. 8-OHdG formation was measured using an OxiSelect™ Oxidative DNA Damage ELISA Kit (Cat No. STA320, from Cell Biolabs Inc.) per manufacturers' instructions. The formation of γH2AX was measured from nuclear extracts by western blotting using H2AX as the input control (3).

Evaluation of mitochondrial function.

Mitochondrial DNA copies. Genomic DNA was extracted from the amygdala tissue using a QIAamp DNA Mini Kit (Qiagen) and the mitochondrial DNA was extracted using the REPLI-g Mitochondrial DNA Kit (Qiagen). The purified DNA was used for the analysis of genomic β-actin (marker of the nuclear gene) and ATP6 (ATP synthase F0 subunit 6, marker of the mitochondrial gene) respectively using the qPCR method mentioned above. The primers for genomic β-actin: forward 5'-acc aca gct gag agg gaa atc -3' and reverse 5'- att gcc gat agt gat gac ctg-3'. The primers for ATP6: forward 5'- tag ggc ttc ttc ccc ata cat -3' and reverse 5'- tta gtg aga tgg ggg ttc ctt-3'. The mitochondrial DNA copies were obtained from relative ATP6 copies that were normalized by β-actin copies using the $^{\Delta\Delta}$CT method.

Intracellular ATP level. The intracellular ATP level was determined using the luciferin/luciferase-induced bioluminescence system. An ATP standard curve was generated at concentrations of 10^{-12}-10^{-3}M. Intracellular ATP levels were calculated and expressed as nmol/mg protein (5).

SOD2 activity assay. SOD2 was obtained from the mitochondrial fraction that was isolated using a Pierce Mitochondria Isolation Kit (Pierce) according to manufacturers' instructions. The successful isolation process was confirmed by the absence of TfR (transferrin receptor) protein in mitochondria section and the absence of COXII (cytochrome c oxidase subunit II) protein in cytosolic fraction by western blots. SOD activity was measured as described previously (6). In brief, a stable $O2^{\cdot-}$ source was generated through the conversion action of XOD (xanthine oxidase) from xanthine and was mixed with chemiluminescent (CL) reagents to achieve a stable light emission. The SOD2 sample injection can scavenge O2.- and the subsequent decrease of chemiluminescent response is proportional to the SOD2 activity. This system can have a detection limit of 0.001U/ml within the linear range of 0.03~2.00U/ml. The results were normalized by protein concentration and expressed as Units/mg proteins (U/mg) (7).

In vivo rat experiments. Sprague Dawley rats were obtained from Guangdong Medical Animal Center. They were maintained under standard 12h light/dark cycles and given ad libitum access to food and water. The animal protocol conformed to US NIH guidelines (Guide for the Care and Use of Laboratory Animals, No. 85-23, revised 1996), and was reviewed and approved by the

Institutional Animal Care and Use Committee from Guangzhou Women and Children Medical Center.

Rat Protocol 1 for generation of diabetic offspring. Adult (3 months old) female Sprague Dawley rats were monitored for estrous cycles with daily vaginal smears. Only rats with at least two regular 4 to 5 day estrous cycles were included in the studies. Chronic diabetic female rats were induced by injection of 50 mg/kg streptozocin (STZ, 0.05 M sodium citrate, pH 5.5) after an 8 hr fasting. Animals with blood glucose >300mg/dl were considered positive, while control (CTL) rats received only vehicle injection. The females were caged with proven males, and the pregnancy was verified through observation of a sperm plug, which was designated as day 0 of pregnancy. The amygdala neurons were isolated on embryonic day 18 (E18) as described below. The male offspring were separated from the dams on day 21 and fed until 7-8 weeks old for further experiments. Some of the 7-8 week-old offspring were then used for autism-like behavior tests. After that, the offspring were sacrificed, and various brain tissues, including the amygdala, hypothalamus and hippocampus, were isolated, flash frozen in dry ice, and then stored in a −80°C freezer for immunohistochemistry, analysis of gene expression, SOD2 activity, superoxide anion release, DNA damage and mitochondrial function.

Rat Protocol 2 for postnatal manipulation of SOD2 expression**.** The male offspring (6 weeks old) from either the CTL or STZ group in Rat Protocol 1 were anesthetized with a mixture of ketamine (90 mg/kg) and xylazine (2.7 mg/kg) and implanted with a guide cannula targeting the amygdala (26 gauge; Plastics One) (8). The following coordinates were chosen for the amygdala: −2.0mm posterior to bregma, ±4.2mm from the midline, and −7.2 mm from the skull surface on which it was based. Cannula was attached to the skull with dental acrylic and jeweler's screws and closed with an obturator (9). An osmotic minipump (Alzet model 2002; flow rate 0.5 μl/h; Cupertino, CA) connected to a 26-gauge internal cannula that extended 1 mm below the guide was implanted and used to deliver SOD2 overexpression (↑SOD2), SOD2 knockdown (shSOD2), or empty (EMP) lentivirus. Vehicle consisting of artificial cerebrospinal fluid (aCSF; 140 mM NaCl, 3 mM KCl, 1.2 mM Na2HPO4, 1 mM MgCl2, 0.27 mM NaH2PO4, 1.2 mMCaCl2, and 7.2 mM dextrose, pH 7.4) was used for the infusion of the lentivirus. Infusion (flow rate 0.5 μl/h) begun immediately after placement of the minipump. 0.5μl of total 2×10^{3} cfu of lentivirus was infused for 1 hour. Rats received the infusion of lentivirus of either SOD2 knockdown or overexpression, or empty control (EMP). The experimental rats were separated into 4 groups (10 per group). Group 1: CTL offspring with empty control lentivirus infusion (CTL/EMP); Group 2: STZ offspring with empty control lentivirus infusion (LNG/EMP); Group 3: STZ offspring with SOD2 expression lentivirus infusion (STZ/↑SOD2); Group 4: CTL offspring with SOD2 knockdown lentivirus infusion (VEH/shSOD2); Cannula placement was verified histologically postmortem by the injection of 0.5μl of India ink (volume matched drug delivery in the experiments). Rats whose dye injections were not located in the amygdala were excluded from the data analysis. 2 weeks after lentivirus infusion, the offspring were used for behavior tests followed by biomedical analysis, as indicated in Rat Protocol 1 (4).

Rat Protocol 3 for prenatal treatment of antioxidants. The verified pregnant dams from either CTL or STZ group in Rat Protocol 1 were randomly assigned to the following 4 groups: Group 1: CTL group rats received only subcutaneously vehicle (5% DMSO in maize oil) injection (CTL/PreVEH); Group 2. Diabetic (STZ) rats received only vehicle injection (STZ/PreVEH); Group 3. Diabetic (STZ) rats received 10mg/kg/day of MnTBAP (dissolved in DMSO) injection (STZ/PreMnTBAP); Group 4. Diabetic (STZ) rats received 20 mg/kg of resveratrol (RSV, dissolved in DMSO) injection (STZ/PreRSV). The injection was conducted on days 1, 4, 7, 10, 15, and 20 of pregnancy, respectively. The male offspring were separated from the dams on day 21 and fed until 7-8 weeks old for behavior tests followed by biomedical analysis.

Rat Protocol 4 for postnatal treatment of antioxidants. The male offspring (3 weeks old) from either the CTL or STZ group in Rat Protocol 1 were randomly assigned to the following 4 groups: Group 1: CTL group rats received only subcutaneous vehicle injection (CTL/PostVEH); Group 2. STZ

rats received only vehicle injection (STZ/PostVEH); Group 3. STZ rats received MnTBAP injection (STZ/PostMnTBAP); Group 4. STZ rats received RSV injection (STZ/PostRSV). The injection was conducted once every 3 days and continuously for 4 weeks, and the rats were sacrificed at 7-8 weeks old for behavior tests followed by biomedical analysis.

Immunohistochemistry. Slides from brain tissues containing amygdala were deparaffinized in Histoclear solution (National Diagnostics) and rehydrated in a series of ethanol. Antigen was retrieved using 10mM sodium citrate buffer (pH 6.0) at 100°C for 10 min, and then treated with 0.3% H2O2. After incubation with 0.3% Triton X-100 in PBS for 15 min and blocking with normal goat serum for 30min, tissues were incubated in primary antibodies SOD2 (#sc-133134, from Santa Cruz Biotechnology) & NeuN (#24307 from Cell Signaling), or ERβ (#sc-390243, from Santa Cruz Biotechnology) & NeuN at 4°C for 12 h, and subsequently with secondary antibodies Alexa Fluor 647 (#ab150083 from Abcam) & Alexa Fluor 488 (#ab150117 from Abcam). Coverslips were examined using a fluorescence microscopy (Leica, 20x lens), and quantitated by Image J. software. For 8-oxo-dG staining, deparaffinized tissue sections were incubated with Proteinase K for 30 min at 37°C, then with buffer containing 100 μg/ml RNase A, 150 mM NaCl and 15 mM sodium citrate for 1h at 37°C. Denature the DNA by treating slides with 2M HCl for 5 min at room temperature, then neutralize the sample by soaking the slides in 1M Tris-base for 5 min at room temperature. After blocking with normal goat serum, incubate sections with 8-oxo-dG anti-mouse antibody (# 4354-MC-050, from Novus Biologicals) for 12h at 4°C and subsequently with secondary antibody Alexa Fluor 488. The cover slips were then mounted by antifade Mountant with DAPI (staining nuclei, in blue). The photographs were taken by using a Confocal Laser Microscope (Leica, 20x lens) and quantitated by Image J. software.

In vitro primary culture of amygdala neurons. Amygdala tissues were dissected from rats on embryonic day 18 (E18 rats). Tissues were treated with 0.05% trypsin EDTA for 15 min at 37°C. Trypsin EDTA was replaced with soybean trypsin inhibitor (Sigma) for 5 min at 37°C to stop the reaction. This was then replaced with supplemented Neurobasal A (Invitrogen) followed by mechanical dissociation. Cells were then resuspended in culture media, including Neurobasal A, B27, 1×GlutaMAX and 100 U/ml Pen/Strep (from Invitrogen), and then the cells were incubated at 37°C, 5% CO2 (10). The isolated amygdala neurons were used for immunostaining, analysis of DNA methylation, and epigenetic changes by ChIP assay (4).

Animal behavior test. The animal behavior test of offspring was carried out at 7-8 weeks of age. Autism-like behavior was evaluated using ultrasonic vocalizations and a three-chambered social test as described below (11-13).
Ultrasonic vocalizations (USVs). The USVs of neonates were examined during brief maternal separation on postnatal day 7. USVs from individually-isolated pups were recorded using an externally polarized condenser microphone with a frequency range of 30-300kHz that was attached 15-20cm above the floor of an isolation chamber. The microphone was connected to the Avisoft-UltrasoundGate recording software (Avisoft Bioacoustics, Germany) and the pup-emitted calls were recorded to WAV sound files using parameters optimized for rats. Pups were individually placed in the sound-proof chambers and calls were recorded for 300s. Data transformation on the number of USVs were analyzed using a generalized linear model with a negative binomial distribution and a log-link function (11, 12).
Social recognition. The social recognition is defined by reduced time spent investigating a familiar conspecific as a result of social habituation, and subsequent reinstatement of investigation when a novel intruder is introduced (dishabituation). Unfamiliar age- and sex-matched intact stimulus rats were placed in wire mesh containers. Before the test, the stimulus rats were gently habituated to being in the container and focal rats were habituated to having an empty container in their home cage. Each focal rat was tested five times (tests 1-5) in their home cage, in which a

container with a stimulus rat was introduced. Each test lasted 5 min and the tests were repeated with a 15-min interval. During the 15-min interval the same empty container was placed back in the home cage of the focal rat. In the first four tests the same stimulus rat was used, whereas for the fifth test, the stimulus rat was replaced with another unfamiliar sex- and age-matched conspecific. The placement of the containers over the five tests was kept constant. During the tests the rats were left undisturbed and their behavior was videotaped and subsequently scored using JWatcher software program. Social investigation was defined as sniffing the wire mesh part of the container (12, 13).

Three-chambered social test. 7-8 week old rats were used to assess sociability and the preference for social novelty. Target subjects (Stranger 1 and Stranger 2) were 7-8 week old rats habituated to being placed inside wire cages for 3 days prior to beginning of testing. Test rats were habituated to the testing room for at least 45 min prior to the start of behavioral tasks. For the sociability test, the test animal was introduced to the middle chamber and left to habituate for 5 min, after which an unfamiliar mouse (Stranger 1) was introduced into a wire cage in one of the side-chambers and an empty wire cage on the other side-chamber. The test animal was allowed to freely explore all 3 chambers over a 10 min session. Following this, a novel stranger rat (Stranger 2) was introduced into the previously empty wire cage and the test animal was again left to explore for a 10 min session. Parameters scored include time spent in each chamber and number of entries into the chambers. Time spent in each chamber and track maps were calculated using the automated SMART software (13).

Immunostaining. The isolated amygdala neurons were transferred to cover slips, and add pre-warmed (37°C) staining solution containing 500nM MitoBeacon™ probe (labeling mitochondria, in red). After 15 min of incubation under growth conditions, the neurons were washed with PBS, fixed in 4% paraformaldehyde for 20 min, and incubated with 0.3% Triton X-100 in PBS for 15 min. After blocking with 5% goat serum in PBS at room temperature for 30 min, neurons were incubated in primary antibody Tuj1 at 4°C for 12 h and subsequently with Alexa Fluor 488 secondary antibody (staining neurons, in green). The cover slips were then mounted with antifade Mountant with DAPI (staining nuclei, in blue). The photographs were taken by using a Confocal Laser Microscope (Leica, 40x lens).

In vivo superoxide anion ($O_2^{\cdot-}$) release. Superoxide anion release from amygdala tissues was determined by a luminol-EDTA-Fe enhanced chemiluminescence (CL) system supplemented with DMSO-TBAC (Dimethyl sulfoxide-tetrabutyl-ammonium chloride) solution for extraction of released $O_2^{\cdot-}$ from tissues, as described previously. The superoxide levels were calculated from the standard curve generated by the xanthine/xanthine oxidase reaction (5).

Statistical analysis. The data was given as mean ± SEM, and all the experiments were performed at least in quadruplicate unless indicated otherwise. The one-way analysis of variance (ANOVA) followed by the Turkey−Kramer test was used to determine statistical significance of different groups, and the two-way ANOVA followed by the Bonferroni post hoc test was used to determine the effect of social recognition by SPSS 22 software, and a *P* value of <0.05 was considered significant.

Table S1. Sequences of primers for the real time quantitative PCR (qPCR)

Gene	Species	Analysis	Forward primer (5'→3')	Reverse primer (5'→3')
β-actin	Human	mRNA	gatgcagaaggagatcactgc	atactcctgcttgctgatcca
Egr1	Human	mRNA	catgatccccgactacctgtt	ctgagtggcaaaggccttaat
ERβ	Human	mRNA	atgatgatgtccctgaccaag	acatcagccccatcattaaca
SOD2	Human	mRNA	gcctacgtgaacaacctgaac	tgaggtttgtccagaaaatgc
SOD2	Human	ChIP	cagcgcaaccaaaactcag	ctgtctgccgtacttgagtgg
β-actin	Rat	mRNA	ttccttcctgggtatggaatc	cttctgcatcctgtcagcaat
ERβ	Rat	mRNA	tcagcatgaagtgcaaaaatg	ggttctgggagctctctttgt
SOD2	Rat	mRNA	caactcaggttgctcttcagc	ctcaaaagacccaaagtcacg

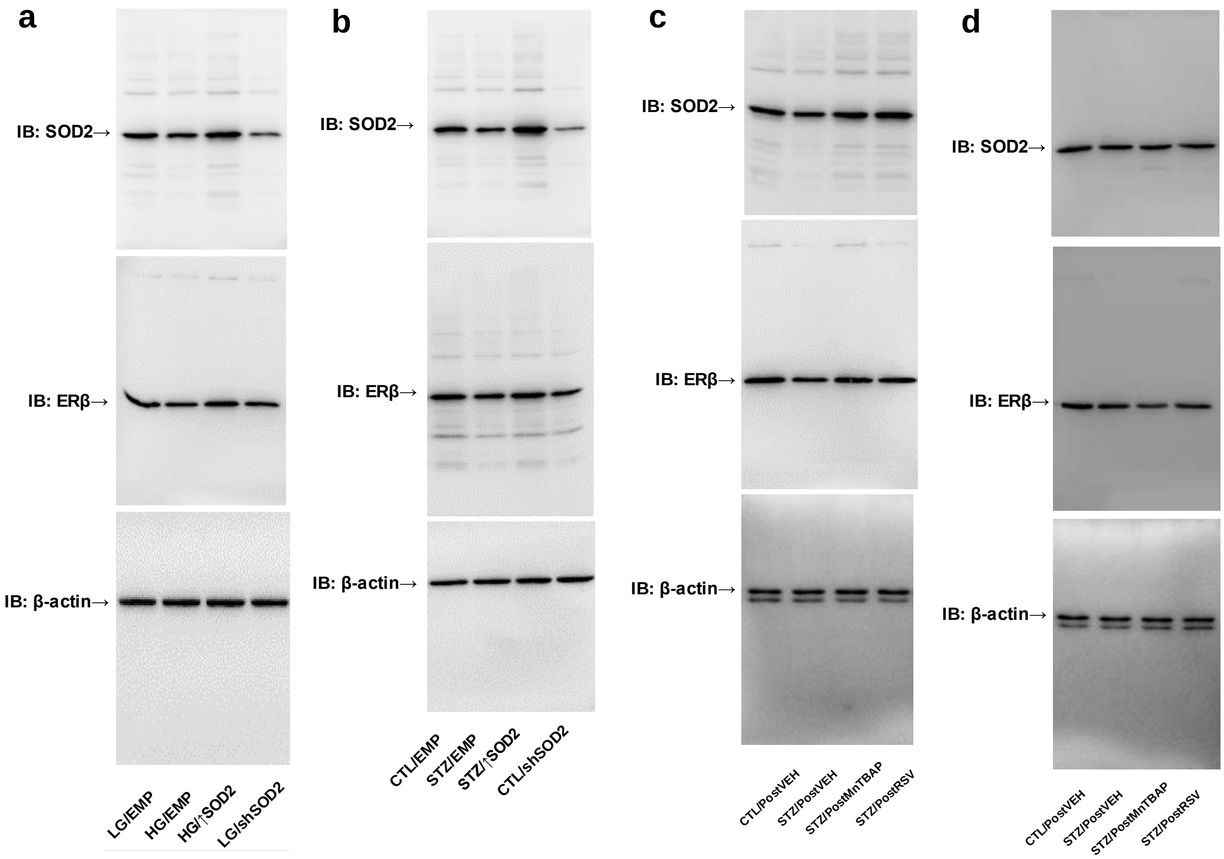

Fig S1. Representative pictures of full blots for Western Blotting. (a) Full blots for Figure 1c. (b) Full blots for Figure 4c. (c). Full blots for Figure 7c. (d) Full blots for Figure 8c.

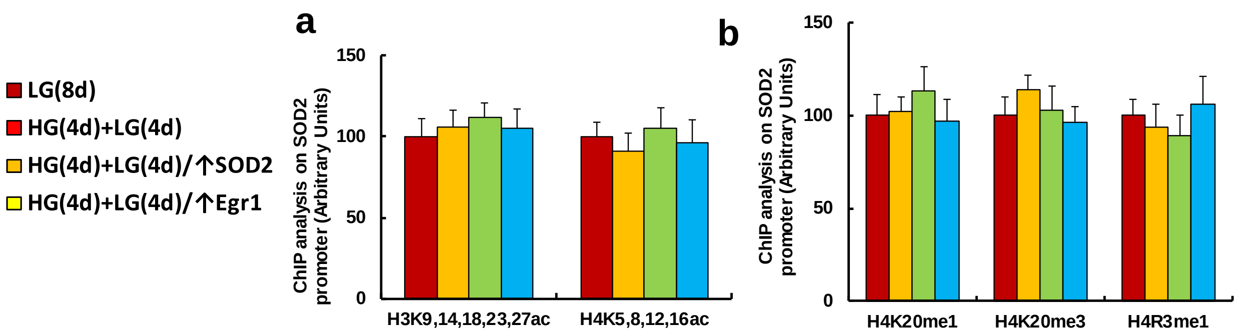

Fig S2. Hyperglycemia-mediated epigenetic changes on the SOD2 promoter. The ACS-5003 neurons were treated by either 8 day LG (LG(8d)), or 4 day HG plus 4 day LG (HG(4d)+LG(4d)), or the cells were infected at day 4 by SOD2 lentivirus (HG(4d)+LG(4d)/↑SOD2), or Egr1 lentivirus (HG(4d)+LG(4d)/↑Egr1), then the cells were used for ChIP analysis. (a) Hyperglycemia-mediated histone acetylation on the SOD2 promoter using H3K9,14,18,23,27ac and H4K5,8,12,16ac antibodies, n=4. (b) Hyperglycemia-mediated histone H4 methylation on the SOD2 promoter, n=4. Data were expressed as mean ± SEM.

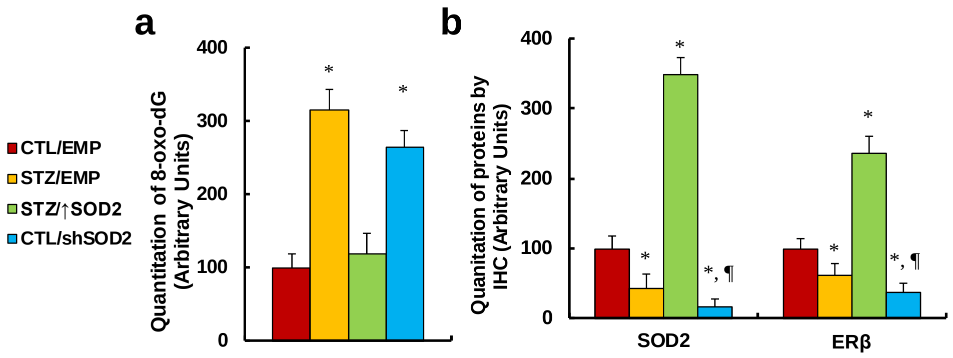

Fig S3. Immunohistochemistry staining of amygdala. The 6-week old male offspring from either control (CTL) or prenatal diabetes (STZ) group received either empty control (EMP), SOD2 overexpression (↑SOD2), or SOD2 knockdown (shERβ) lentivirus infusion, then the offspring at 8-week old were sacrificed, and the amygdala was isolated for immunohistochemistry staining. (a) Quantitation of 8-oxo-dG staining, n=5. (b) Quantitation of SOD2 and ERβ staining, n=5. *, $P<0.05$, vs CTL/EMP group; ¶, $P<0.05$, vs STZ/EMP group. Data were expressed as mean ± SEM.

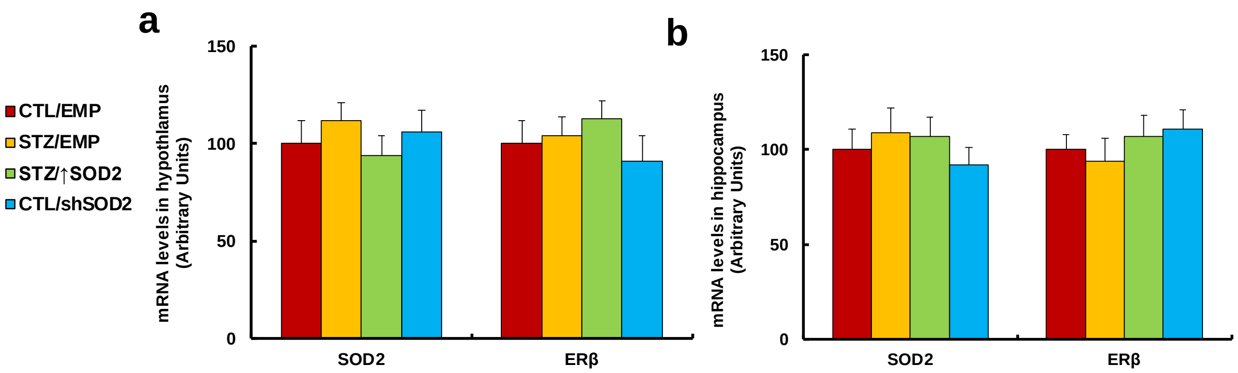

Fig S4. Prenatal diabetes does not affect the gene expression of SOD2 and ERβ in the hypothalamus and hippocampus. The 6-week old male offspring from either control (CTL) or prenatal diabetes (STZ) group received either empty control (EMP), SOD2 overexpression (↑SOD2), or SOD2 knockdown (shERβ) lentivirus infusion, then the offspring at 8-week old were sacrificed, and the hypothalamus and hippocampus were isolated for gene expression analysis. (a) The mRNA levels in hypothalamus, n=4. (b) The mRNA levels in hippocampus, n=4. Data were expressed as mean ± SEM.

REFERENCES

1. Bodnar AG, Ouellette M, Frolkis M, Holt SE, Chiu CP, Morin GB, Harley CB, Shay JW, Lichtsteiner S, and Wright WE. Extension of life-span by introduction of telomerase into normal human cells. *Science.* 1998;279(5349):349-52.
2. Kong D, Zhan Y, Liu Z, Ding T, Li M, Yu H, Zhang L, Li H, Luo A, Zhang D, et al. SIRT1-mediated ERbeta suppression in the endothelium contributes to vascular aging. *Aging Cell.* 2016;15(6):1092-102.
3. Zhang H, Li L, Li M, Huang X, Xie W, Xiang W, and Yao P. Combination of betulinic acid and chidamide inhibits acute myeloid leukemia by suppression of the HIF1alpha pathway and generation of reactive oxygen species. *Oncotarget.* 2017;8(55):94743-58.
4. Zou Y, Lu Q, Zheng D, Chu Z, Liu Z, Chen H, Ruan Q, Ge X, Zhang Z, Wang X, et al. Prenatal levonorgestrel exposure induces autism-like behavior in offspring through ERbeta suppression in the amygdala. *Mol Autism.* 2017;8(46.
5. Yao D, Shi W, Gou Y, Zhou X, Yee Aw T, Zhou Y, and Liu Z. Fatty acid-mediated intracellular iron translocation: a synergistic mechanism of oxidative injury. *Free Radic Biol Med.* 2005;39(10):1385-98.
6. Yao D, Vlessidis AG, Gou Y, Zhou X, Zhou Y, and Evmiridis NP. Chemiluminescence detection of superoxide anion release and superoxide dismutase activity: modulation effect of Pulsatilla chinensis. *Anal Bioanal Chem.* 2004;379(1):171-7.
7. Kong D, Zhan Y, Liu Z, Ding T, Li M, Yu H, Zhang L, Li H, Luo A, Zhang D, et al. SIRT1-mediated ERbeta suppression in the endothelium contributes to vascular aging. *Aging Cell.* 2016.
8. Neal-Perry G, Yao D, Shu J, Sun Y, and Etgen AM. Insulin-like growth factor-I regulates LH release by modulation of kisspeptin and NMDA-mediated neurotransmission in young and middle-aged female rats. *Endocrinology.* 2014;155(5):1827-37.
9. Hu M, Richard JE, Maliqueo M, Kokosar M, Fornes R, Benrick A, Jansson T, Ohlsson C, Wu X, Skibicka KP, et al. Maternal testosterone exposure increases anxiety-like behavior and impacts the limbic system in the offspring. *Proc Natl Acad Sci U S A.* 2015;112(46):14348-53.
10. Hay CW, Shanley L, Davidson S, Cowie P, Lear M, McGuffin P, Riedel G, McEwan IJ, and MacKenzie A. Functional effects of polymorphisms on glucocorticoid receptor modulation of human anxiogenic substance-P gene promoter activity in primary amygdala neurones. *Psychoneuroendocrinology.* 2014;47(43-55.
11. Silverman JL, Yang M, Lord C, and Crawley JN. Behavioural phenotyping assays for mouse models of autism. *Nat Rev Neurosci.* 2010;11(7):490-502.
12. Schaafsma SM, Gagnidze K, Reyes A, Norstedt N, Mansson K, Francis K, and Pfaff DW. Sex-specific gene-environment interactions underlying ASD-like behaviors. *Proc Natl Acad Sci U S A.* 2017;114(6):1383-8.
13. Moy SS, Nadler JJ, Perez A, Barbaro RP, Johns JM, Magnuson TR, Piven J, and Crawley JN. Sociability and preference for social novelty in five inbred strains: an approach to assess autistic-like behavior in mice. *Genes Brain Behav.* 2004;3(5):287-302.
